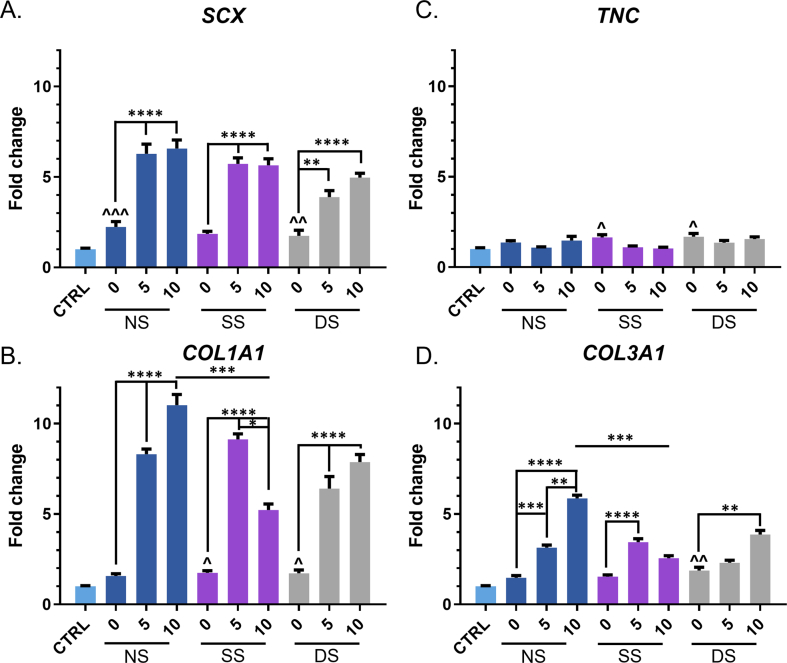# A systematic investigation of the effects of TGF-β3 and mechanical stimulation on tenogenic differentiation of mesenchymal stromal cells in a poly(ethylene glycol)/gelatin-based hydrogel

**DOI:** 10.1016/j.jot.2023.09.006

**Published:** 2023-10-21

**Authors:** Ilze Donderwinkel, Rocky S. Tuan, Neil R. Cameron, Jessica E. Frith

**Affiliations:** a Department of Materials Science and Engineering, Monash University, Clayton, VIC, 3800, Australia; b Institute for Tissue Engineering and Regenerative Medicine, The Chinese University of Hong Kong, Hong Kong SAR, China; c School of Engineering, University of Warwick, Coventry, CV4 7AL, United Kingdom; d Australian Research Council Training Centre for Cell and Tissue Engineering Technologies, Monash University, Clayton, VIC, 3800, Australia; e Australian Regenerative Medicine Institute, Monash University, Clayton, VIC, 3800, Australia

**Keywords:** Biochemical stimulation, Hydrogels, Mesenchymal stromal cells, Physical stimulation, Tendon tissue engineering, Tenogenic differentiation

## Abstract

**Background:**

High post-surgical failure rates following tendon injury generate high medical costs and poor patient recovery. Cell-based tendon tissue engineering has the potential to produce fully functional replacement tissue and provide new strategies to restore tendon function and healing. In this endeavour, the application of mesenchymal stromal cells (MSCs) encapsulated in biomaterial scaffolds has shown great promise. However, a consensus on optimal promotion of tenogenic differentiation of MSCs has yet to be reached, although growth factors and mechanical cues are generally acknowledged as important factors.

**Methods:**

In this study, we prepared a hydrogel cell culture system consisting of methacrylated poly(d,l-lactic acid-ethylene glycol-d,l-lactic acid) (P(LA-EG-LA)) and gelatin methacrylate (GelMA) to encapsulate human bone marrow-derived MSCs (hBMSCs). We further systematically investigated the influence of static and intermittent cyclic uniaxial strain mechanical stimulation, in combination with transforming growth factor-β3 (TGF-β3) supplementation, on tenogenic differentiation of hBMSCs.

**Results:**

Increased TGF-β3 concentration upregulated the tenogenic genes Scleraxis (*SCX)* and collagen type I (*COL1A1)* but showed no effects on tenascin-c (*TNC)* and collagen type III (*COL3A1)* expression. Mechanical stimulation had no observable effect on gene expression, but intermittent cyclic uniaxial strain stimulation improved matrix deposition. Together, these data provide new insights into how TGF-β3 and mechanical stimulation regulate MSC tenogenesis, with TGF-β3 promoting the expression of key tenogenic genes whilst mechanical stimulation aided matrix deposition in the engineered tissue. Furthermore, intermittent cyclic uniaxial strain at 3% elongation and 0.33 ​Hz for 1 ​h/day showed improved matrix effects compared to static strain.

**Conclusion:**

Together, the most promising result for tenogenic differentiation of hBMSCs was identified as treatment with 5 ​ng/ml TGF-β3 under intermittent cyclic uniaxial strain (3% strain; 0.33 ​Hz; 1 ​h/day). This knowledge is of importance for the development of an improved protocol for tenogenic differentiation of MSCs and thereby for tendon tissue engineering.

**The translational potential of this article:**

Tissue-engineered strategies for tendon repair require a consensus on the differentiation of mesenchymal stromal cells to tenocytes, which is currently lacking. This article provides a systematic investigation of two main tenogenic differentiation conditions to further development of a tenogenic differentiation protocol.

## Graphical abstract

**Figure:**
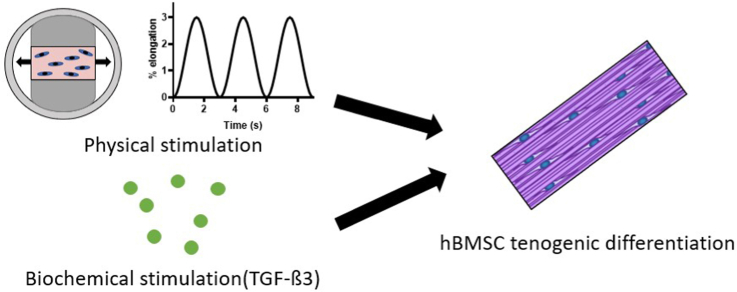

## 1 Introduction

Tendon and ligament-related injuries lead to 30 million surgical procedures annually and mainly affect the athletic and elderly populations [1]. Patients with tendon ruptures or tendinopathy suffer from constant pain and restricted use of the affected tendon, which affects their mobility. Following damage, the tendon tissue goes through the natural healing process characterised by inflammatory, fibroblastic/proliferative and matrix remodelling stages [2]. A hallmark of tendon repair is that Transforming Growth Factor (TGF) β1 induces myofibroblast differentiation leading to disorganised collagen deposition and creating scar tissue [3,4]. Differences in the composition and structure of the repair tissue, as compared to native tendon, contribute to its reduced mechanical properties, leading to frequent tearing. Thus, even with surgical intervention, full functionality of the ruptured tendon is not restored, and outcomes are hampered by unacceptably high failure rates [5].

New strategies are therefore required to reduce failure rates and restore tendon function. Tendon tissue engineering aims to regenerate the native tendon tissue by combining artificial scaffolds with cells to produce fully functional tendon tissue. Adult human tissue-derived mesenchymal stromal cells (MSCs) have shown great potential due to their availability, low immunogenicity and ability to generate tenogenic cells [1]. However, a major challenge in the tendon tissue engineering field is the current lack of a consensus on how to promote tenogenic differentiation of MSCs [6]. Mechanical stimulation is an important factor for tenogenic differentiation of MSCs [7,8] and can be applied via static or dynamic means, with particular divergence in the methods for dynamic stimulation where a variety of commercially-available or bespoke bioreactors are used [7,9,10]. A recent study showed both an increase in tenogenic gene expression under static and cyclic uniaxial loading with the statically loaded samples showing improved cell alignment and cyclic loaded samples showing improved protein content [11]. Cyclic strain was furthermore shown to regulate collagen isoforms in fibrillogenesis [12]. Moreover, a substantial number of growth factors are also being studied for their effect on tenogenic differentiation. Among these, TGF-β3 is widely acknowledged as one of the key driving growth factors for tenogenic initiation [[13], [14], [15]], but its optimal concentration has not yet been established. A major limitation is that few studies systematically compare the effects of different physical stimulation types on MSC tenogenesis when combined with biochemical stimulation. To engineer a native-like tendon tissue, it is therefore essential to determine and optimize the interplay between biochemical and physical stimulation of MSCs, as well as the differences between static or dynamic stimulation.

In this study, we systematically compare combinations of TGF-β3 and varying types of mechanical stimulation on the tenogenic differentiation of human bone-marrow derived MSCs (hBMSCs) in a poly(ethylene glycol) (PEG) and gelatin-based hydrogel cell culture system. The suitability of the hydrogels for cell encapsulation was confirmed by cell viability, spreading and proliferation. Furthermore, the mechanical properties and robustness of the hydrogels were explored to verify stability during intermittent cyclic uniaxial strain mechanical stimulation. Hydrogel-encapsulated hBMSCs were stimulated using either static or intermittent cyclic uniaxial mechanical stimulation, together with TGF-β3 - a growth factor naturally expressed during foetal tendon healing where no scar tissue is formed [16]. Tenogenesis was evaluated based on tenogenic marker gene expression and histology. The findings in this paper provide insights into the interplay between biochemical and mechanical stimulation and add to the understanding of tenogenesis in hBMSCs, providing valuable knowledge for tendon tissue engineering.

## 2 Materials & methods

Chemicals were purchased from Sigma–Aldrich (Castle Hill, NSW, Australia) unless stated otherwise. Human bone marrow-derived mesenchymal stromal cells (hBMSCs) from three donors (Donor 1: 34Y,F; Donor 2: 23Y,F; Donor 3: 26Y,F) were purchased from Lonza (Mt Waverley, VIC, Australia). Low glucose cell culture medium was supplied by Life Technologies, USA (Dulbecco's Modified Eagle's Medium (DMEM), containing d-glucose and sodium pyruvate). Life Technologies, UK; Scientifix Life, USA; and Lonza, AU supplied, respectively, penicillin-streptomycin 100 U ​ml^−1^ (pen/strep), foetal bovine serum (FBS), and Transforming Growth Factor-β3 (TGF-β3). Dulbecco's Phosphate-buffered saline (DPBS) was purchased from Thermo Fisher (Scoresby, VIC, Australia). All experiments were performed with biological and technical triplicates.

### 2.1 Synthesis of methacrylated P(DLLA-EG-DLLA)

Tin(II) 2-ethylhexanoate (0.7 eq) was added to a Schlenk tube equipped with stirring bar and dried under high vacuum for 24 ​h while stirring at 350 ​rpm. PEG-OH with a MW of 4000 ​g/mol (1 eq) and 3,6-dimethyl-1,4-dioxane-2,5-dione (4 eq) were added while under N_2_ gas. The Schlenk tube was then placed in a preheated oil bath (135 ​°C) and left to stir at 350 ​rpm. After 24 ​h the reaction was cooled to room temperature and full conversion was checked using FTIR. A minimum amount of dichloromethane was added to dissolve the polymer, which was then precipitated in ice-cold isopropanol. The isopropanol mixture was kept at −20 ​°C overnight to fully precipitate the polymer, which was obtained by filtration of the mixture, and left to air dry for 2.5 ​h before vacuum drying for 2 days. NMR analysis was performed to confirm P(DLLA-EG-DLLA) synthesis. The obtained polymer (1 eq) was dissolved in dichloromethane and placed in a Schlenk tube equipped with stirring bar. Dry triethylamine (3 eq) and methacrylic anhydride (3 eq) were added to the mixture and left to stir at 500 ​rpm at room temperature for 7 days while under Ar gas. The reaction mixture was precipitated in ice-cold diethyl ether and left overnight at −20 ​°C to fully precipitate out. The solution was filtered and washed with ice-cold diethyl ether. The residue was redissolved in a minimum amount of chloroform and reprecipitated in ice-cold diethyl ether. The solution was filtered after 3 ​h at −20 ​°C and the residue was vacuum dried for 2 days. The obtained white product was analysed by NMR spectroscopy (Bruker DPX 300) and MALDI spectroscopy (Bruker MALDI ToF MS AutoFlex III), confirming synthesis of P(LA_2_-EG_91_-LA_2_)-bMA.

### 2.2 Methacrylation of gelatin

Gelatin type A (G1890, Sigma–Aldrich) was dissolved at 10 ​wt% in carbonate-bicarbonate (CB) buffer, 0.25 ​M, pH 9. The solution was placed in a preheated oil bath at 50 ​°C until the gelatin was fully dissolved. Methacrylic anhydride (0.08 ​ml per gram of gelatin) was added whilst stirring at 750 ​rpm. The reaction was left to stir for 3 ​h. The reaction was terminated by readjusting the pH to 7.4. The reaction mixture was diluted with warm water and transferred to dialysis tubes (MWCO ​= ​14 ​kDa). The product was lyophilized after dialysis for 3 days against MilliQ water at 40 ​°C changing water every 3–4 ​h. A white solid was obtained and stored at −20 ​°C. The obtained product was characterized using NMR spectroscopy. The degree of substitution (DS) was determined using the Habeeb method (DS ​= ​75%) [17].

### 2.3 Cell culture

hBMSCs (Lonza) were cultured in low glucose cell culture medium (LG-DMEM) supplemented with 1% (v/v) pen/strep and 10% (v/v) FBS and incubated in humidified conditions at 37 ​°C with 5% CO_2_. Medium was refreshed every 3–4 days and cells were passaged at 80% confluence and split at a cell density of 2–2.5 ​× ​10^3^ ​cells/cm^2^. To detach cells, they were washed with DPBS preceding trypsinization with TrypLE for 3–5 ​min at 37 ​°C. Cell culture medium containing FBS was added to deactivate enzymatic digestion. Cell numbers were determined by using a haemocytometer. hBMSCs were used at passage 6 for experimental purposes unless stated otherwise.

### 2.4 Hydrogel preparation and cell encapsulation

Fresh stock solutions of GelMA 20% (w/v), P(LA_2_-EG_91_-LA_2_)-bMA 20% (w/v), and lithium phenyl-2,4,6-trimethylbenzoylphosphinate (LAP) 5% (w/v) were prepared in LG-DMEM. For cell encapsulation, LG-DMEM was replaced by LG-DMEM with pen/strep and FBS supplementation. Trypsinized hBMSCs were pelleted at 1200 ​rpm for 3 ​min and resuspended in the GelMA stock solution to reach a final cell concentration of 2.5 ​× ​10^6^ ​cells/ml in the hydrogel construct. For all constructs the following procedure was followed. Calculated amounts of stock solutions were added to LG-DMEM to reach a final concentration of 8 ​wt% GelMA, 2 ​wt% P(LA_2_-EG_91_-LA_2_)-bMA, and 0.3 ​wt% LAP. Hydrogel precursor solutions were loaded to a mould or on a tissue culture plate and gelation occurred upon photopolymerisation using visible light (λ ​= ​400–500 ​nm, 10 ​mW/cm^2^, 10 ​min, Omnicure S2000, Lumen dynamics). Upon full gelation, hydrogel constructs were immersed in cell culture medium or DPBS and incubated in humidified conditions at 37 ​°C and 5% CO_2_.

### 2.5 Hydrogel characterization and degradation

For degradation studies, 50 ​μl hydrogels were prepared as droplets or linear constructs. Initial weight was recorded as m_t0_ before submerging in DPBS and incubation at 37 ​°C. At each time point, hydrogels were removed from the solution and excess solution was disposed using a Kimwipe. Hydrogel mass was recorded as m_t_ at each time point and placed in fresh DPBS. Degradation was calculated as the percentage of m_t_ to the initial mass m_t0_: $m_{t}/m_{t0}\times 100\%$.

To study swelling and gel fraction, hydrogel weight was recorded as m_p_ before photopolymerisation. After light irradiation, constructs were swollen for 24 ​h in an excessive amount of DPBS in which solution was refreshed regularly to remove any uncured polymer. The fully swollen gel mass was recorded as m_s_. Hydrogels were then lyophilized and the dry weight was recorded as m_d_. Percentage swelling was calculated as $\left(m_{s}/m_{p}\right)/m_{p}\times 100\%$, equilibrium water content percentage (EWC) as $\left(m_{s}-m_{d}\right)/m_{s}\times 100\%$, and gel fraction as $m_{d}/m_{p}\times 100\%$.

### 2.6 Rheology

Oscillatory rheological analysis was performed on hydrogels (8 ​wt% GelMA, 2 ​wt% P(LA_2_-EG_91_-LA_2_)-bMA, 0.3 ​wt% LAP in LG-DMEM) pre- and post-swelling on a parallel plate strain-controlled rheometer (Anton Paar Physica). Time sweeps were performed at 37 ​°C with a 0.3 ​mm gap between a 15 ​mm plate and quartz glass bottom plate. Paraffin oil was applied on air-exposed gel to limit water evaporation during measurement. Loaded gels (75 ​μl) were allowed to stabilize for 2 ​min before light exposure (λ ​= ​400–500 ​nm, 10 ​mW/cm^2^, Omnicure S2000, Lumen dynamics), with amplitude (0.5%) and frequency (0.16 ​Hz) kept constant. All measurements were taken in triplicate.

### 2.7 Cell viability assay

Cell viability was determined using a Live/Dead assay kit (Life Technologies, Scoresby, VIC, Australia) as per manufacturer's instructions. Briefly, hBMSCs were encapsulated in hydrogels and transferred at the appropriate timepoint to DPBS for 15 ​min to limit undesirable serum esterase activity. Staining solution containing 2 ​μM calcein AM (0.05% v/v) and 4 ​μM EtD-1 (0.2% v/v) was prepared in sterile DPBS. Hydrogel constructs were submerged in the staining solution and incubated for 30 ​min at room temperature in the dark. Cells were imaged at three predetermined areas per sample using a Nikon Eclipse Ts2 Inverted Routine Microscope equipped with a Lumenera Infinity3-3UR camera using Infinity analysis software. Each condition was imaged in triplicate and analysed using ImageJ software (NIH, USA) by manual counting of live and dead cells.

### 2.8 Immunofluorescence

Hydrogels were incubated in DPBS for 15 ​min and fixed in 4% buffered paraformaldehyde (PFA) for 30 ​min at room temperature. Constructs were then washed in PBS three times. To diminish hydrogel autofluorescence, hydrogels were pre-stained with 0.1% (w/v) Sudan Black B (Astral Scientific, Taren Point, NSW, Australia) in 70% (v/v) ethanol for 20 ​min at room temperature. After three washes in DPBS supplemented with 0.02% (v/v) Tween 20, cell permeabilisation was carried out with 0.5% (v/v) Triton X-100 in DPBS for 20 ​min. After DPBS washing, non-specific blocking was done for 1 ​h at room temperature in 3% (w/v) bovine serum albumin (BSA). The hydrogel constructs were then incubated in primary antibody (anti-Ki67; ab15580, 1:750; anti-collagen I, ab34710, 1:100; Abcam, Melbourne, VIC, Australia) in 3% BSA overnight at 4 ​°C in a humidified chamber. On the following day, after three DPBS washes, the constructs were incubated for 1 ​h at room temperature in 3% (w/v) BSA containing secondary antibody (goat anti-rabbit AF488, A11008, Life Technologies, 1:1000), and for cell morphology with ActinRed (R37112, Life Technologies, 2 drops/ml) and Hoechst33342 (H3570, Life Technologies, 1:1000). Constructs were washed five times in DPBS before imaging at 3–5 predetermined areas per sample using an Inverted Routine Microscope (Nikon Eclipse Ts2) or 3–5 predetermined areas per sample in z-stack mode on a confocal microscope (Nikon A1Rsi, Japan). Images were analysed using ImageJ software (NIH, USA) and NIS elements viewer software (Nikon, Japan). Collagen deposition was quantified as intensity divided by cell number to obtain collagen intensity per cell.

### 2.9 Trough gel preparation

In order to run the static and dynamic set-up simultaneously, trough gels were prepared with a similar dimension as the Tissue Train® trough loaders 6-well posts. A linear Tissue Train® culture plate (Flexcell Int Corp, Burlington, NC, USA) was silanized using trichloro(1H,1H,2H,2H-perfluorooctyl)silane in a desiccator overnight. In a clean Styrofoam cup, silicone elastomer base and curing agent from a Sylgard (Dow Corning) Industrial 184 Silicone Elastomer Kit (Revolution Industrial, Seaford, VIC, Australia) were mixed in a 10:1 ratio and stirred to assure mixing. The polydimethylsiloxane (PDMS) precursor solution was then degassed in a desiccator for 30–60 ​min. The silanized Tissue Train® culture plate was put into the Flexcell FX-4000T Tension System (Flexcell Int Corp, USA) using the Tissue Train® trough loaders 6-well posts and put under a vacuum upon which the PDMS precursor solution was added and left to gel for at least 24 ​h. Upon vacuum release, inverted PDMS moulds were then taken out of the linear trough culture plate, silanized and sterilized using 80% (v/v) ethanol. PDMS precursor solution was repeated and added to a sterile 6-well plate (Corning) to which the inverted PDMS moulds were added. The gel was allowed to set overnight in a 60 ​°C oven. The inverted PDMS moulds were taken out of the wells, leaving a trough imprint in the below PDMS. The tissue culture plate containing PDMS trough moulds was then further sterilised using 80% (v/v) ethanol and dried in a 60 ​°C oven for 1 ​h. Plates were used for cell culture once returned to room temperature.

### 2.10 Tenogenic differentiation

Cyclic mechanical stimulation for tenogenic differentiation of hBMSCs was applied by the use of a Flexcell FX-4000T Tension System using Linear Tissue Train® culture plates (Flexcell Int Corp, USA). Vacuum was applied over Trough Loaders 6-well posts for hydrogel loading between the anchor stems using a hydrogel volume of 50 μl/well. Upon gelation, the vacuum was released, and cell culture medium was added. Hydrogel constructs containing cells were left to stabilize for 24 ​h. Cell culture medium was then refreshed and supplemented with 5 ​ng/ml or 10 ​ng/ml TGF-β3. The Trough Loaders 6-well posts were replaced by Arctangle Loading stations 6-well posts and the constructs were subjected to intermittent cyclic uniaxial strain at 3% elongation and 0.33 ​Hz for 1 ​h/day. For static mechanical stimulation, hydrogel (50 ​μl) was deposited between two pins at each end of the PDMS trough mould, and the constructs were treated the same as for cyclic mechanical stimulation. Medium was replaced every 3–4 days and supplemented with TGF-β3. Hydrogel constructs were retrieved at day 7 post-encapsulation, washed in DPBS for 15 ​min, and processed for analysis.

### 2.11 Analysis of gene expression by quantitative real time polymerase chain reaction (qRT-PCR)

Pooled triplicate technical replicates were processed into small pieces and cells were retrieved by addition of a minimum amount of trypsin–EDTA (0.5%, Life Technologies). Samples were incubated at 37 ​°C until the hydrogel had fully dissolved. DPBS was added and samples were spun down for 4 ​min. The remaining pellet was subsequently resuspended in 300 ​μl QIAzol lysis reagent (Qiagen). RNA isolation and purification was performed using a Qiagen RNeasy mini kit upon which RNA quantity was determined spectrophotometrically using a Nanodrop (DeNovix DS-11 FX) and RNA quality assessed based on A_260/280_. SuperScript VILO Master Mix (Life Technologies, Australia) was used to synthesize cDNA for qRT-PCR per manufacturer's instructions. Target cDNA was amplified using SYBR Green Reaction Mixture (Applied Biosystems, Thermo Fisher Scientific) using a Bio-Rad T100 Thermal Cycler. Gene expression levels of scleraxis (SCX), tenascin-c (TNC), collagen type I (COL1A1) and collagen type III (COL3A1) were quantified and normalized to ribosomal protein 27a (RPS27a) using the –ddCT method to calculate fold changes, with dCt values used for statistics **(**Table 1**)**.

**Table 1:** Sequences of qRT-PCR primers.

Gene	Forward primer (5′ to 3′)	Reverse primer (5′ to 3′)	NCBI accession number
RPS27a	TGGATGAGAATGGCAAAATTAGTC	CACCCCAGCACCACATTCA	BC066293
SCX	TGCGAATCGCTGTCTTTC	GAGAACACCCAGCCCAAA	XM_006716616
TNC	GGTGGATGGATTGTGTTCCTGAGA	CTGTGTCCTTGTCAAAGGTGGAGA	NM_002160
COL1A1	CCTGCGTGTACCCCACTCA	ACCAGACATGCCTCTTGTCCTT	NM_001845
COL3A1	CAGCGGTTCTCCAGGCAAGG	CTCCAGTGATCCCAGCAATCCC	NM_000090

### 2.12 Histology

Samples for histological analysis were fixed using 4% PFA for 30 ​min and washed three times in DPBS. Samples were subsequently transferred to cryomoulds (Tissue-Tek, ProSciTech, Kirwan, QLD, Australia), incubated in 30% BSA overnight and embedded in OCT compound (Tissue-Tek). Cryo-embedding of samples was performed using 2-methylbutane and liquid nitrogen. Sections of 5 ​μm were prepared using a Leica CM3050 cryostat at −23 ​°C. Sections were defrosted for 30 ​min at room temperature before staining with haematoxylin & eosin (H&E), Alcian Blue, Picrosirius Red or Masson's trichrome.

### 2.13 Statistics

Statistical analysis was performed on triplicate measurements and presented as mean ​± ​standard deviation (SD) unless stated otherwise. GraphPad Prism 8 was used to check for normality and equal variance before performing statistical analysis using a one-tailed t-test to test two groups, a one-way ANOVA followed by Tukey multiple comparison test to test three groups or more, or a two-way ANOVA to test multiple donor effects. Statistical analysis for qRT-PCR was performed on dCT values of pooled technical triplicates for each donor. Statistical significance was set at p ​< ​0.05 (ns, p ​> ​0.05; ∗, p ​≤ ​0.05; ∗∗, p ​≤ ​0.01; ∗∗∗, p ​≤ ​0.001).

## 3 Results

### 3.1 Polymer synthesis and functionalisation

A hydrogel was developed as a 3D culture system for the encapsulation and differentiation of hBMSCs, that could further be used as a biodegradable scaffold. The hydrogel should be non-cytotoxic, support cell proliferation and spreading, and have the mechanical strength to withstand any forces exerted by the mechanical stimulation used to induce tenogenesis. P(LA-EG-LA) has previously been used as the biomaterial for a biodegradable, biocompatible and physiologically strong hydrogel [18], whereas gelatin can be used to enhance cell function [19]. Therefore, we opted to develop a combined hydrogel, synthesised by crosslinking methyacrylated P(LA-EG-LA) and gelatin. ABA triblock copolymers of P(LA-EG-LA) were synthesized by ring opening polymerisation of d,l-lactide initiated by PEG (Supplementary Fig. 1A). Successful polymerisation on both PEG hydroxyl groups was confirmed by NMR spectroscopy upon emergence of a peak at δ4.2–4.4 ​ppm corresponding to methylene protons (b, Supplementary Fig. 1B) with PLA block length calculations, using the integrals of d and b,c, indicating a polymer composition of P(LA_2_-EG_91_-LA_2_) (Supplementary Fig. 1B). The hydroxyl end groups of the P(LA-EG-LA) were then methacrylated to obtain methacrylic-functionalised P(LA_2_-EG_91_-LA_2_) polymers, (P(LA_2_-EG_91_-LA_2_)-bMA), suitable for crosslinking. Methacrylation was confirmed by NMR spectroscopy corresponding to the methyl group at *δ* 1.96 ​ppm (g), and methylene group at *δ* 5.62 ​ppm and *δ* 6.20 ​ppm (f,f’) and also verified using MALDI spectroscopy which showed an increase in molecular weight between P(LA_2_-EG_91_-LA_2_) and P(LA_2_-EG_91_-LA_2_)-bMA (Supplementary Figs. 1B and C).

Porcine type A gelatin was methacrylated to facilitate crosslinking with P(LA_2_-EG_91_-LA_2_)-bMA. NMR spectroscopy confirmed methacrylation of the gelatin, as determined by a decrease in the lysine methylene proton peak at *δ* 2.84–2.94 ​ppm and appearance of the methacrylate methyl proton peak at *δ* 1.86 ​ppm and methacrylate methylene proton peaks at *δ* 5.37 ​ppm and *δ* 5.60 ​ppm (Supplementary Fig. 1D). The degree of substitution (DS) was determined to be 75% by the Habeeb method [17] confirming the successful functionalisation of gelatin for further crosslinking with P(LA_2_-EG_91_-LA_2_)-bMA.

### 3.2 Hydrogel fabrication and characterisation

Photo-crosslinked P(LA_2_-EG_91_-LA_2_)-bMA (2 ​wt%) and GelMA (8 ​wt%) hydrogels were produced using LAP as a photoinitiator (0.3 ​wt%) under visible light (λ ​= ​400–500 ​nm). Oscillatory rheological analysis showed that gelation occurred within 30 ​s of light exposure with a stable storage modulus (G’) being reached after approximately 8 ​min at 1930 ​± ​230 ​Pa for pre-swelled gels and 200 ​± ​40 ​Pa for post-swelled gels (Fig. 1A). Swelling studies revealed an equilibrium water content (EWC) of 93.9 ​± ​0.2% 24 ​h post-swelling, with a 16.5 ​± ​0.4 swelling ratio and gel fraction of 67.8 ​± ​7.0, which is comparable to other PEG/gelatin-based hydrogels [20].

**Figure 1:**
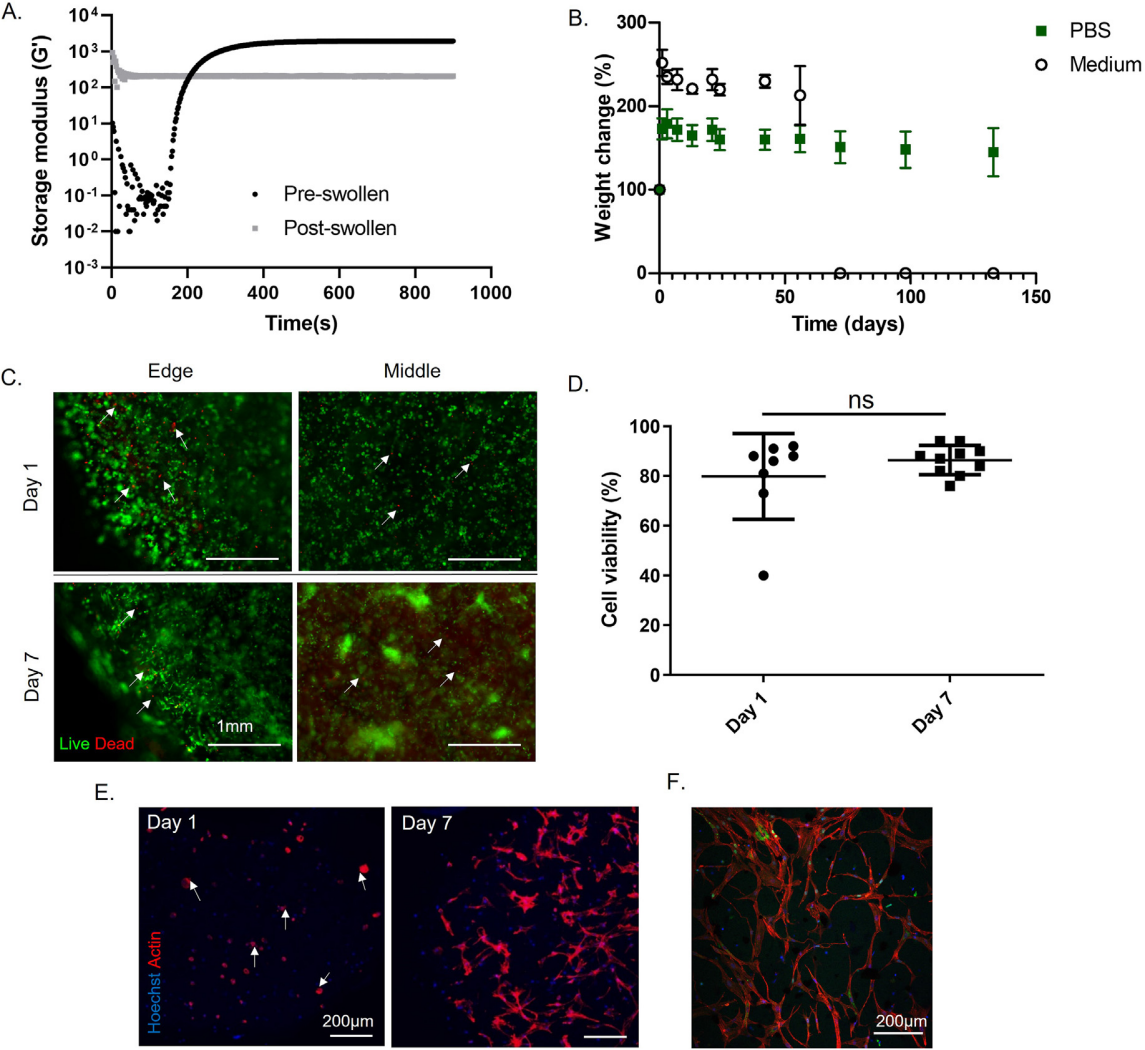
Hydrogel characterisation. (A) Oscillatory photorheological measurements of storage modulus (G′) pre-swelling (black) and post-swelling (grey). (B) Degradation of linear non-stimulated hydrogels by weight change over time in DPBS (green squares) and cell culture medium (open circles) at 37 ​°C. (C–D) Live/Dead staining (C) and quantitation (D) of hBMSCs encapsulated in hydrogels at 1 day and 7 days post-encapsulation. Scale bar ​= ​1 ​mm. (E) Cell spreading examined by F-actin filament (red) and Hoechst (blue) staining at day 1 and day 7. Arrows indicate spherical cells with small filopodia. (F) Ki67 staining (green) of MSCs at day 7. Scale bars ​= ​200 ​μm. Graphs are shown as mean ​± ​SD, N ​= ​3; ns, p ​> ​0.05; ∗, p ​< ​0.05; ∗∗, p ​< ​0.01; ∗∗∗, p ​< ​0.001. (For interpretation of the references to color in this figure legend, the reader is referred to the Web version of this article.)

The degradation profile of the hydrogel was monitored by incubating samples in either DPBS or cell culture medium in the absence of cells at 37 ​°C. Noting that hydrogel surface area and geometry have an influence on degradation, comparisons were made between hydrogel droplets and a linear hydrogel with dimensions matching those required for later tenogenesis experiments and integration into the Flexcell FX 400T Tension System (Fig. 1B; Supplementary Fig. 2). All hydrogels increased in weight after 24 ​h due to water absorption as anticipated by the swelling ratio (Fig. 1B; Supplementary Fig. 2). Notably, the constructs in cell culture medium increased in weight to a greater extent (252 ​± ​27%) than constructs in DPBS (173 ​± ​22%), likely due to the higher degree of swelling caused by a higher concentration of salts available in the cell culture medium. When assessing degradation, the linear constructs in DPBS started to decrease in weight from day 56 and remained intact up to 133 days whilst those in cell culture medium showed a rapid decrease in weight after 42 days and full degradation by day 72 (Fig. 1B). The faster degradation in cell culture medium is potentially due to the supplements in culture medium, but in any case, these constructs were still stable for a sufficient period that they would be expected to support tenogenic differentiation of MSCs *in vitro* [11,21].

To confirm the viability of cells in the conditions required for further tenogenic studies, Live/Dead staining was performed on hBMSCs encapsulated and cultured within the linear hydrogel constructs. At one day post-encapsulation a high cell viability of 82 ​± ​7% was observed, which increased after 7 days of culture to 86 ​± ​1% (Fig. 1C and D). This confirmed the non-toxic effect of both the hydrogel precursors, the process of making the linear constructs, and the ability of the hydrogel scaffold to support cell viability. Staining for F-actin filaments was carried out to assess the ability of hBMSCs to spread within the hydrogel. At day one post-encapsulation hBMSCs appeared mostly spherical with small filopodia (Fig. 1E, denoted by arrows). After a week in culture, cell spreading was prominent throughout the entire gel confirming that sufficient integrin-binding motifs remained in the gelatin after methacrylation (Fig. 1E). Cell proliferation was assessed using the proliferation marker Ki67 to mark actively cycling cells [22]. Ki67-positive cells were present in cells throughout the constructs confirming the ability of the hydrogels to support cell proliferation (Fig. 1F). Quantitation of the percentage of Ki67-positive cells supported this, although with varying levels of proliferation according to the donor (Supplementary Fig. 3, donor 1: 0.5 ​± ​0.01%; donor 2: 15.1 ​± ​5.4%; donor 3: 9.5 ​± ​5.6%).

### 3.3 Testing interplay of TGF-β3 provision and mechanical stimulation

Three regimes were developed to determine the impact of mechanical stimulation on tenogenesis (Supplementary Fig. 4A). Linear hydrogels cultured under standard conditions in well plate (non-stimulated, NS) were compared to those anchored at either end by minutien pins to passively create tension across the construct (static stimulation, SS) and also to hydrogels exposed to intermittent cyclic uniaxial strain (dynamic stimulation, DS) using the Flexcell FX-4000T Tension System. This uses a vacuum to dynamically pull a flexible membrane over a static post thereby stretching the loaded construct secured by two anchor points. A regimen of 3% sinusoidal cyclic uniaxial strain at 0.33 ​Hz for 1 ​h/day was used based on the optimal conditions found by Youngstrom et al.*,* Jones et al., and Kuo and Tuan [7,8,23]. Importantly, histological evaluation of acellular constructs after 24h continuous stimulation confirmed that the hydrogels were robust enough to withstand the applied forces as evidenced by a lack of any damage in samples stained with Toluidine blue (Supplementary Fig. 4B).

To test the interplay of different TGF-β3 concentrations together with these three different mechanical stimulation regimes, constructs under each of these mechanical conditions were cultured in the absence of additional TGF-β3 provision, or in culture medium supplemented with 5 ​ng/ml or 10 ​ng/ml TGF-β3. Conditions will from hereon be abbreviated by their stimulation type followed by the TGF-β3 concentration; for example, the non-stimulated control in absence of TGF-β3 is sample NS-0. After 7 days culture, the viability of the encapsulated hBMSCs was assessed via Live/Dead staining and showed cell viabilities of over 80% for all conditions (Fig. 2A). Haematoxylin and Eosin staining of sections through the hydrogels was also used to evaluate the cellular distribution and structure of the constructs (Fig. 2B). Irrespective of the stimulation conditions, this showed cells distributed throughout the hydrogels but with a thicker layer of more elongated cells on the edge of the gel (Fig. 2B and C; Supplementary Fig. 8). The cells within the gels were evident within small cavities in the hydrogel matrix, often in small clusters (Fig. 2C).

**Figure 2:**
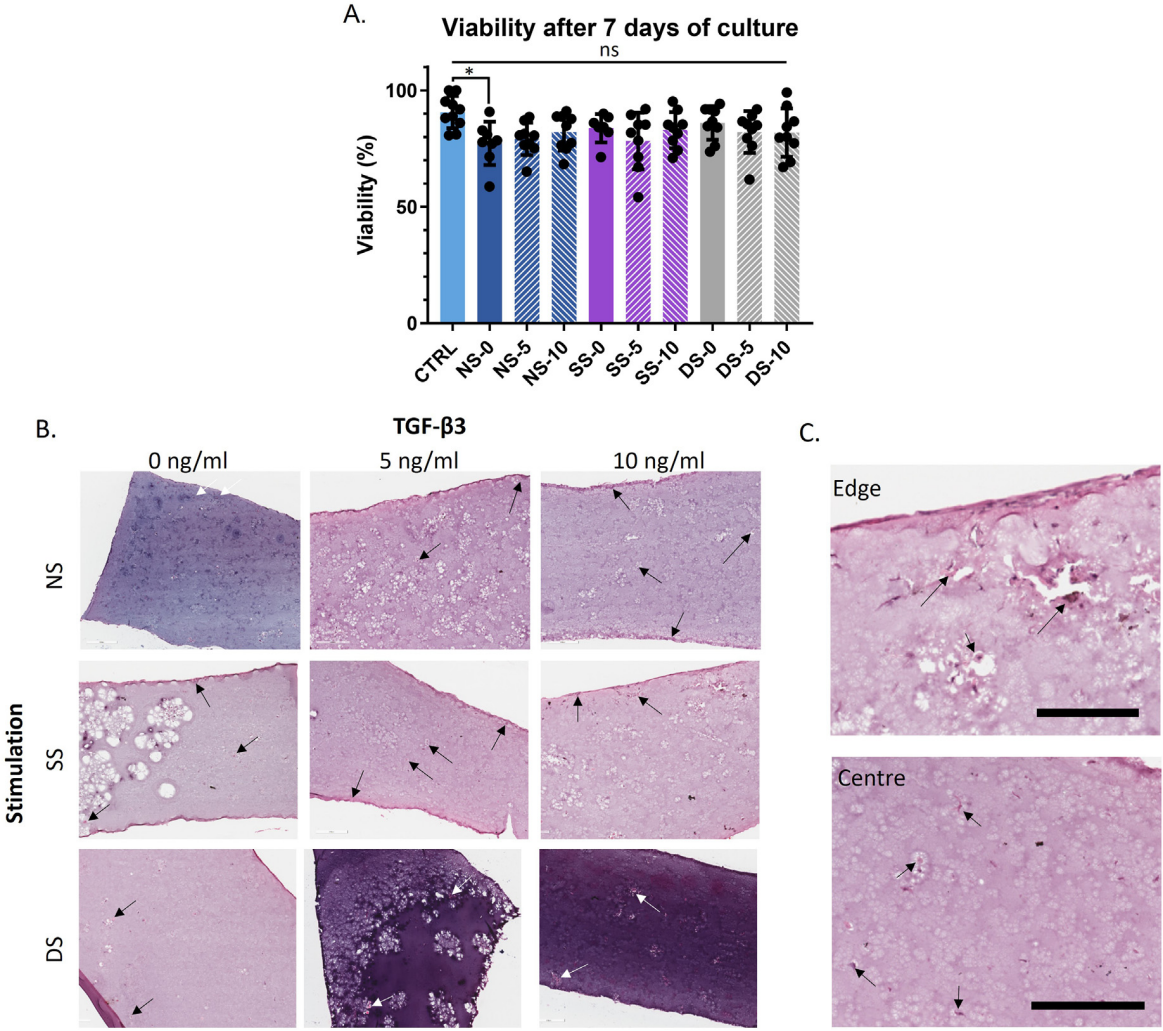
Evaluation of cell viability and distribution in tenogenic constructs. (A) Graph showing the proportion of viable cells in the varying constructs after 7 days culture. Graphs are shown as mean ​± ​SD, N ​= ​3; ns, p ​> ​0.05; ∗, p ​< ​0.05; ∗∗, p ​< ​0.01; ∗∗∗, p ​< ​0.001. (B) H&E staining of sections through the tenogenic constructs after 7 days culture. (C) High magnification images of the edge and centre of an SS-10 construct. Cell nuclei are indicated by arrows. Scale bars ​= ​200 ​μm. NS ​= ​non-stimulated, SS ​= ​static stimulation, DS ​= ​dynamic simulation.

### 3.4 Effect of TGF-β3 and mechanical stimulation on tenogenic gene expression

**Figure 3:**
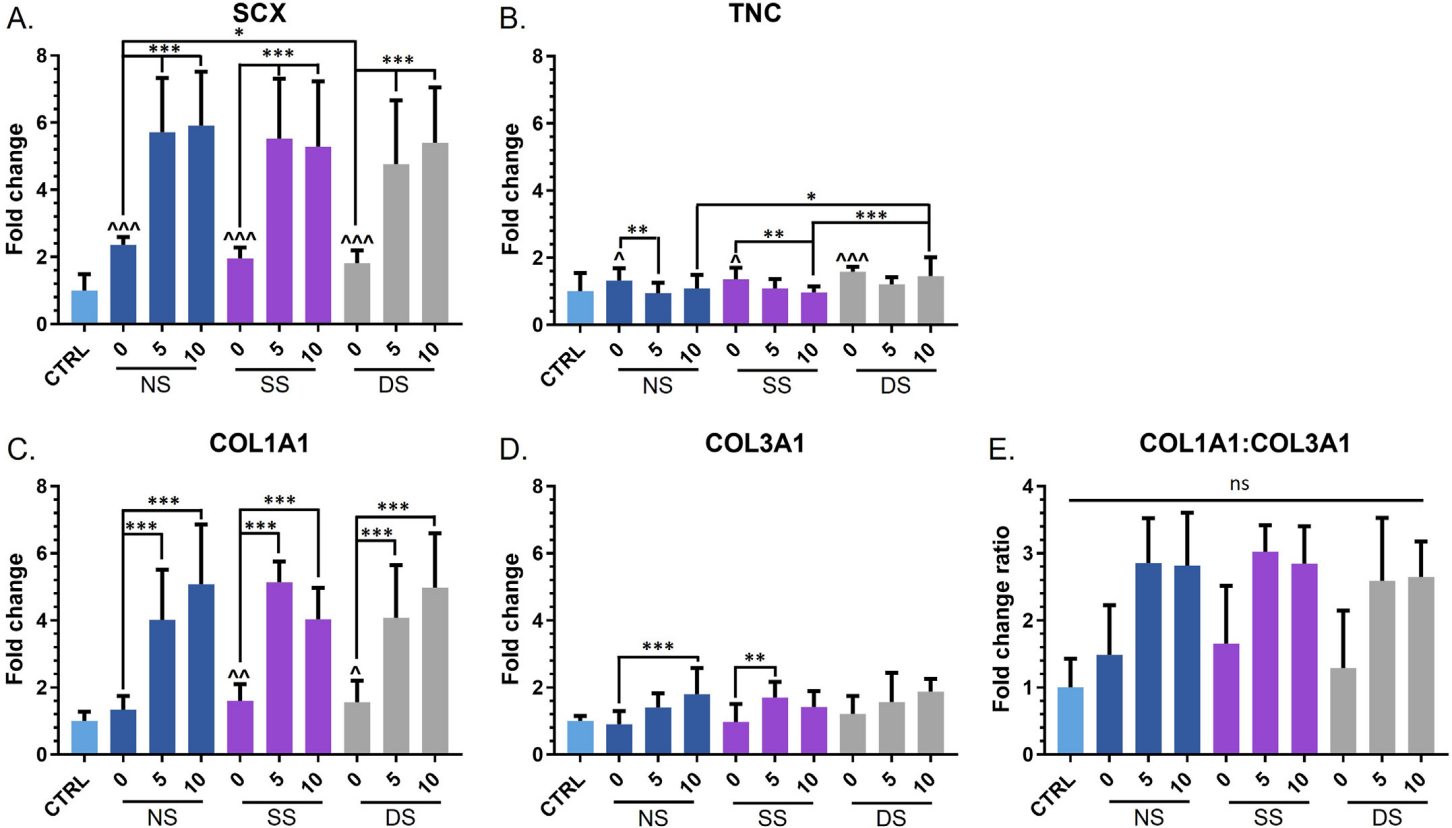
Tenogenic gene expression of (A) scleraxis (SCX), (B) tenascin c (TNC), (C) collagen type I (COL1A1), and (D) collagen type III (COL3A1) in hBMSC-hydrogel constructs as a function of TGF-β3 treatment dose and mechanical stimulation after culture day 7. Analysis was performed on hBMSCs encapsulated in droplet (CTRL) or linear hydrogels for 7 days without stimulation (NS), or under static (SS) or dynamic (DS) mechanical stimulation, in the absence (0) or presence of 5 ​ng/ml (5) or 10 ​ng/ml (10) TGF-β3. (E) Gene expression ratio of COL1A1 to COL3A1. Graphs are shown as mean ​± ​SD, N ​= ​3; ns, p ​> ​0.05; ∗, p ​< ​0.05; ∗∗, p ​< ​0.01; ∗∗∗, p ​< ​0.001. Significant differences to the CTRL are shown in ^.

The impact of the various tenogenic regimes on the expression of key tenogenic marker genes was evaluated by RT-qPCR, specifically measuring the expression of *SCX*, *TNC*, *COL1* and *COL3* (Fig. 3). These were normalised to hBMSCs cultured inside a hydrogel droplet sample without mechanical or TGF-β3 stimulation (CTRL) to also determine whether there were any positive effects from the elongated linear shape of the hydrogels. SCX is currently regarded as the most specific tenogenic and ligament developmental marker available [7], and is expressed from early tenogenesis right through tendon development. When compared to undifferentiated hBMSCs in the CTRL sample, expression of *SCX* increased across all conditions confirming induction of tenogenesis (Fig. 3A). Interestingly, the levels of *SCX* were influenced by the shape of the hydrogel alone where a significant increase in expression was observed in the linear hydrogels compared to CTRL, even in the absence of TGF-β3 or any mechanical simulation (CTRL vs NS-0). When comparing NS-0 to SS-0 and DS-0 no further increase was caused by the static or dynamic stimulation, although a slight decrease could be observed between NS-0 and DS-0. Provision of TGF-β3 substantially enhanced SCX levels at both 10 ​ng/ml and 5 ​ng/ml. No substantial advantage was gained with the higher concentration of TGF-β3, for example with an average 5.7-fold and 5.9-fold increase in NS-5 and NS-10 samples as compared to CTRL.

Expression of the key tenogenic extracellular matrix (ECM) genes, *COL1A1*, *COL3A1* and *TNC* was also monitored. COL1 is the most abundant ECM component of tendon, *COL3A1* is the second most prominent ECM component in tendon, while TNC is involved in collagen fibril organisation and has a highly regulated expression which can be altered by mechanical stress [24]. Changes to the expression of *TNC* were minimal, reaching a maximum of 1.6-fold compared to CTRL for DS-0 (Fig. 3B). Interestingly, DS-10 showed an significant increase compared to NS-10 and SS-10, but not to the control. The overall small changes meant that there was no clear effect of either TGF-β3 or mechanical stimulation, although both static and dynamic stimulation induced a small (∼1.4-fold) increase in levels for Donor 3 which had the greatest tenogenic response of those tested (Supplementary Fig. 7).

Expression of *COL1A1* followed a similar trend to that of *SCX,* in that mechanical stimulation made little difference to the level of expression whereas TGF-β3 caused significant upregulation. Again, no statistically significant advantage was seen in using the higher concentration of 10 ​ng/ml compared to 5 ​ng/ml TGF-β3 (Fig. 3C). *COL1A1* expression was highest overall in the SS-5 samples, with a ∼5.1-fold increase compared to CTRL, followed closely by the DS-10 samples which showed a ∼5.0-fold increase in *COL1A1* (Fig. 3C). When assessing *COL3A1* expression the high variability in magnitude of change between donors meant that little overall statistically significant response was evident. When looking at individual donors, the donor that showed the strongest induction overall (donor 3) did have higher *COL3A1* expression in the presence of TGF-β3, in a manner that was decreased by mechanical stimulation (Fig. 3D; Supplementary Figs. 5–7). However, there was some indication of a positive effect of TGF-β3, with a statistical increase between NS-0 and NS-10, and SS-0 and SS-5 (Fig. 3D). A high COL1:COL3 ratio is preferred in tendon fibrillogenesis considering the native tendon composition and the involvement of collagen type III in scar tissue formation. From this perspective, a non-significant increase in COL1:COL3 ratio can be observed with TGF-β3 supplementation. Overall, these data suggest that TGF-β3 supplementation had a greater effect on the induction of tendon-related gene expression than mechanical stimulation.

### 3.5 Effects of TGF-β3 and mechanical stimulation on ECM deposition and structure

**Figure 4:**
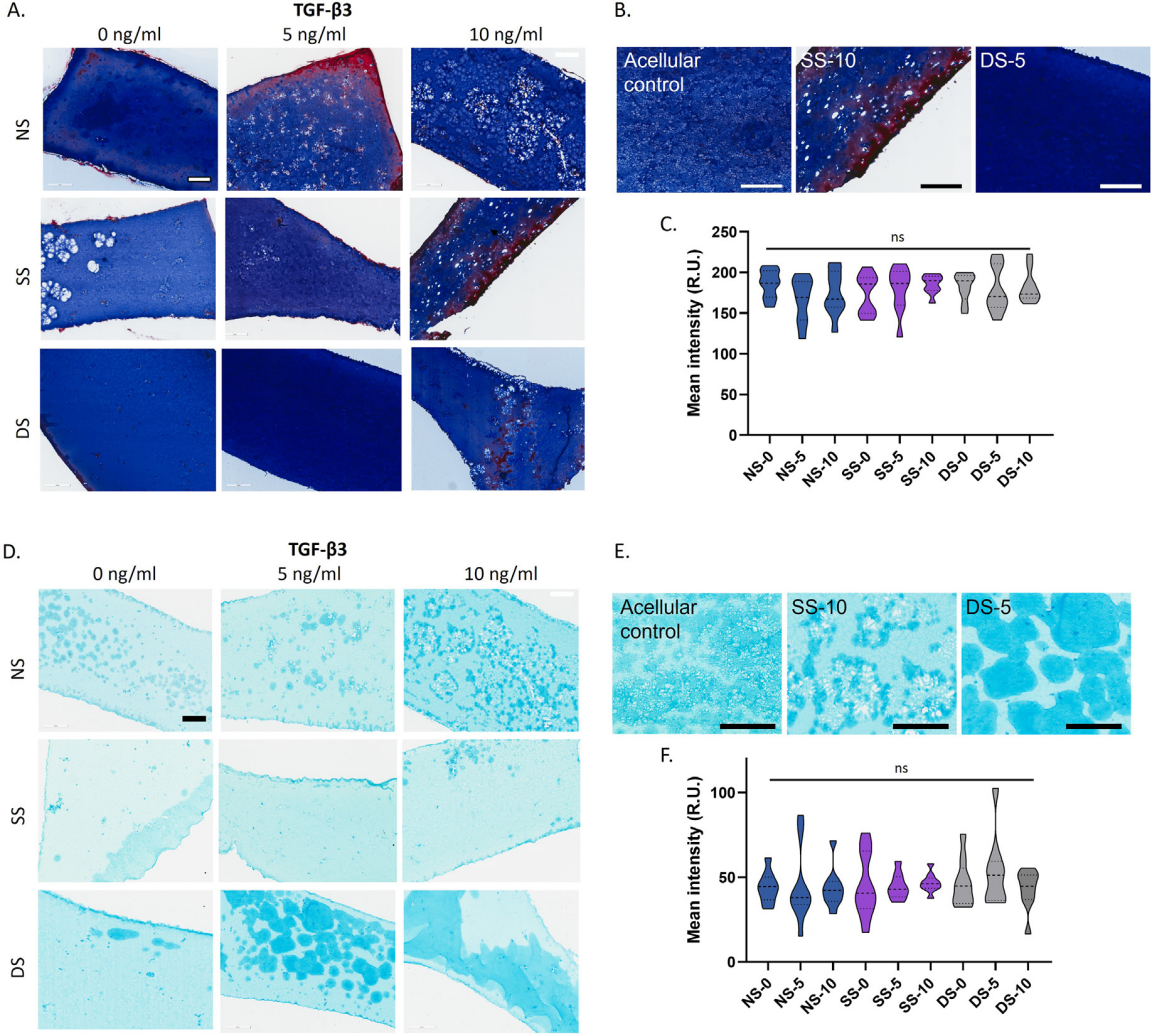
Histological analysis by Masson's trichrome (A–C) and Alcian Blue staining (D–F) of the effects of TGF-β3 treatment dose and mechanical stimulation on hBMSC-hydrogel constructs on culture day 7. (A and D) Representative images of stained sections. (B and E) Higher magnification images. All scale bars ​= ​200 ​μm. (C and F) Violin plots of mean staining intensity for whole sections of triplicates for each biological and technical replicate. N ​= ​3; ns, p ​> ​0.05; ∗, p ​< ​0.05; ∗∗, p ​< ​0.01; ∗∗∗, p ​< ​0.001. (For interpretation of the references to color in this figure legend, the reader is referred to the Web version of this article.)

To further understand whether the combinations of TGF-β3 and mechanical stimulation might affect the deposition and organisation of ECM, histological sections of the day 7 tenogenic constructs were stained with Masson's Trichrome, where collagens stain in blue, muscle and cytoplasm stain red, and nuclei in dark brown/black (Fig. 4A–C). For all histological staining, a cell-free hydrogel construct was maintained in parallel for 7 days and used to determine levels of background staining, with some degree of colouration evident for the Masson's trichrome, likely due to the presence of gelatin in the hydrogel (Supplementary Fig. 10). The overall degree of Masson's trichrome staining was similar across all the conditions regardless of TGF-β3 concentration or mechanical stimulation, as confirmed by image quantitation (Fig. 4C, Supplementary Fig. 9). However, differences in the staining distribution were observed, where the staining of samples subjected to dynamic stimulation was more evenly distributed than non-stimulated or static stimulation constructs (Fig. 4A, Supplementary Fig. 10). Interestingly, multiple sections showed areas of intense red staining, for example for SS-10 (Fig. 4A and B). Red staining was also observed in glutaraldehyde-crosslinked tendon sections following *in vivo* analysis [25]. The authors hypothesized this was due to increased fibril density following crosslinking which inhibited differential penetration of the Aniline Blue and Biebrich scarlet-acid fuchsin stains in Masson's trichrome. Considering this, Aniline Blue and Biebrich scarlet-acid fuchsin staining did not occur properly for these sections as the red staining was not observed for the other donors (Supplementary Fig. 10).

The sections were also stained with Alcian Blue at pH 2.5 to mark sulphated glycosaminoglycans (GAGs), such as decorin and aggrecan (Fig. 4D–F) [26,27]. These are expressed early in tendon development and play an important role in collagen fibrillogenesis [28]. For example, Decorin is homogenously expressed in early tendon development, followed by localization around the cells [29]. The acellular control showed a pale background level of staining with some darker areas present in the centre of the sample (Supplementary Fig. 11). However, the cellular samples had a clear pattern of pericellular localization of the staining which, in many cases, was most prevalent at the periphery of the section, likely due to the abundant presence of highly aligned cells at the edge of the hydrogel (Fig. 4D and E). Quantitation of the staining intensity showed considerable variation between replicates, but with a greater number of sections with high Alcian blue intensity in the NS-5 and DS-5 samples (Fig. 4F, Supplementary Fig. 9). When assessing the impact of mechanical stimulation, static stimulation showed minimal effect on GAG deposition, whilst large circular areas of GAG deposition were observed in samples under dynamic stimulation. In particular, these Alcian blue-positive deposits were numerous in samples under dynamic stimulation and exposed to increasing TGF-β3 concentrations, resulting in substantial areas of GAG-rich matrix (Fig. 4D, Supplementary Fig. 11).

### 3.6 Effects of TGF-β3 and mechanical stimulation on collagen deposition and organisation

**Figure 5:**
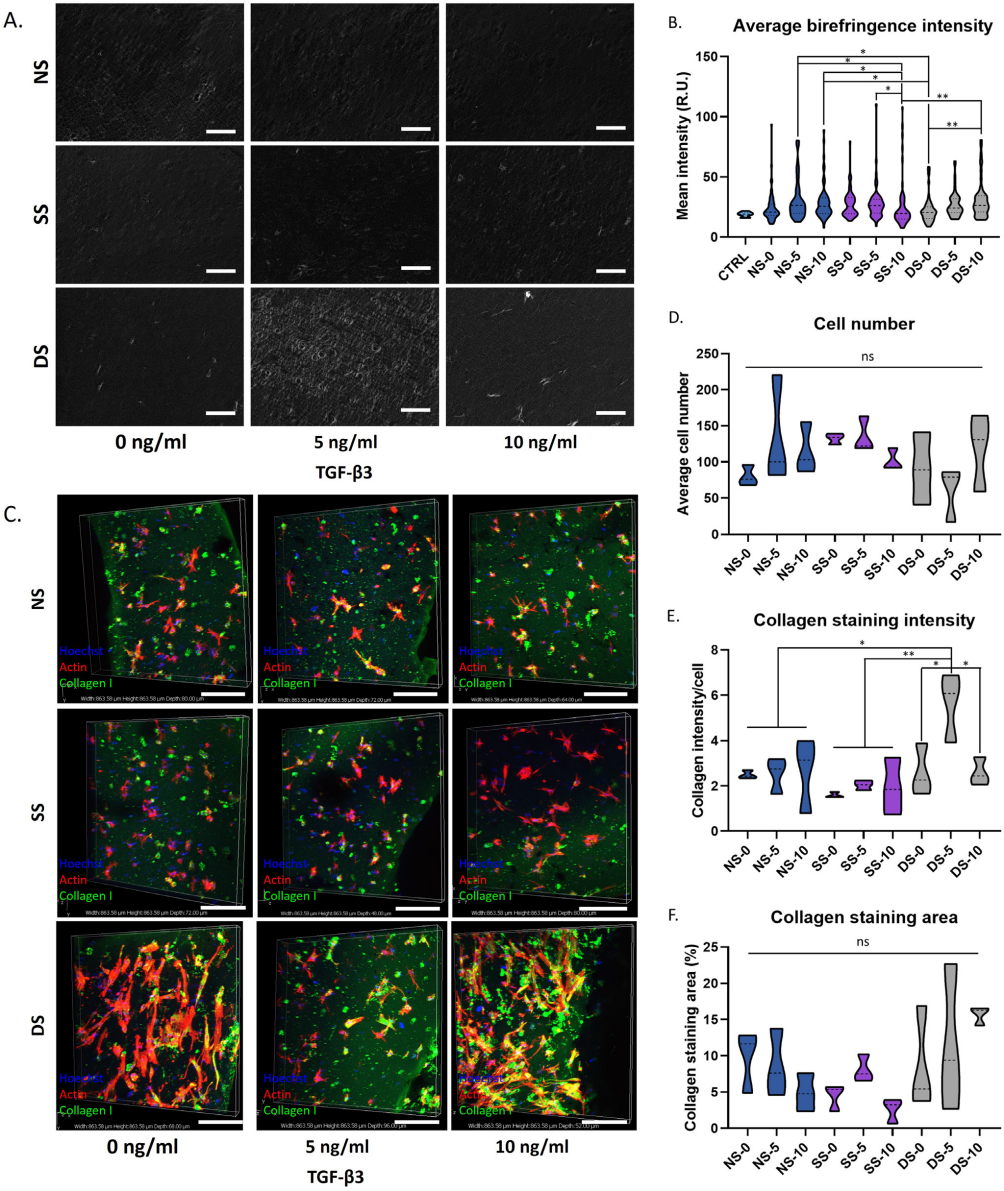
Analysis of collagen deposition in hBMSC-hydrogel constructs on culture day 7 as a function of TGF-β3 treatment dose and mechanical stimulation. (A) Birefringence microscopy images of picrosirius red stained sections near the surface of the hydrogel for Donor 1. Collagen fibres appear white against the grey background. Scale bar ​= ​100 ​μm. (B) Quantitation of birefringence microscopy image intensity of triplicate measures of three biological and technical replicates. (C) Immunohistochemical staining for collagen type I showing collagen type I (green), nuclei (blue), and actin filaments (red) for Donor 1. Scale bar ​= ​200 ​μm. (D–E) Average staining intensity for Donor 1 as measured by ImageJ (E) including average cell number counted (D). (F) Average staining area of collagen type I over total area of two biological and three technical replicates. Graphs are shown as truncated violin plots; ns, p ​> ​0.05; ∗, p ​< ​0.05; ∗∗, p ​< ​0.01; ∗∗∗, p ​< ​0.001. (For interpretation of the references to color in this figure legend, the reader is referred to the Web version of this article.)

To examine the collagen content and distribution more closely, sections were stained with Picrosirius red (Supplementary Figs. 9 and 12) and examined using birefringence microscopy (Fig. 5, Supplementary Figs. 13–15), which permits visualisation of collagen fibre orientation and microstructure within the hydrogel [30]. Throughout all of the samples the collagen fibres were evident in the lighter patches (Fig. 5A) showing a network of fine, often aligned fibres running throughout the construct. Many samples also showed circles reinforced with collagen around the edge of voids in the hydrogel structure, whilst several of the samples under dynamic stimulation showed bright areas of intense collagen around cells encapsulated within the matrix. Samples under dynamic stimulation often had a different structure with areas of collagen fibres around the periphery of the encapsulated cells. Quantitation of the image intensity showed that DS-5 and DS-10 had a generally higher birefringence intensity, which varied in magnitude between MSC donors but was statistically significant for Donor 1 which generally produced higher levels of ECM (Fig. 5B, Supplementary Figs. 13–15).

Immunohistochemistry for collagen type I also showed deposition of collagen type I under all tenogenic conditions (Fig. 5C, Supplementary Fig. 16). Similar amounts were present in the NS and SS samples, but dynamic stimulation resulted in greater collagen staining which increased further at higher TGF-β3 concentrations. The F-actin and Hoechst staining suggested that this might be due to a difference in cell density (Fig. 5C and D); thus, collagen deposition was further quantified as intensity divided by cell number to obtain collagen intensity per cell (Fig. 5E). This showed that while collagen-I deposition was most pronounced under dynamic stimulation, the highest production of collagen:cell was in the DS-5 hydrogels, while the overall staining area did not show any significant difference (Fig. 5E and F). Mean intensity of collagen fibril formation showed a positive effect of mechanical stimulation over TGF-β3 supplementation in all three donors **(**Supplementary Figs. 13–15**)**. Overall, these data suggest dynamic stimulation has a positive influence on collagen-I deposition and fibrillogenesis, with optimal collagen-I deposition in combination with 5 ​ng/ml TGF-β3 supplementation.

## 4 Discussion

Mechanical and biochemical stimulation both greatly influence tendon development and healing. Understanding their interplay is therefore vital in advancing tendon TE. To provide a hydrogel system with suitable properties for cell encapsulation and tenogenesis, it was essential to use a hydrogel that was non-cytotoxic, biodegradable to allow neo-tissue formation, with adequate stiffness and able to withstand strain application. To achieve this, we developed a hydrogel based on crosslinking of methacrylated P(LA-EG-LA) and gelatin, where the synthetic polymer, P(LA-EG-LA), would improve mechanical properties, whilst the integrin-binding motifs and matrix metalloproteinase-sensitive degradation sites in the gelatin would support cell adhesion and matrix turnover [19]. Synthesis of P(LA-EG-LA) as the synthetic polymer was targeted because block copolymers of PLA and PEG are known for their biocompatibility and biodegradability [31].

Overall, the P(LA-EG-LA) and gelatin co-polymer biomaterial produced a hydrogel scaffold that was mechanically robust and biocompatible to hBMSCs. Rheological analysis showed an appropriate storage modulus post-swelling to support even distribution of cells during encapsulation for even matrix deposition. Mechanical testing further showed mechanical robustness at forces required for dynamic stimulation while degradation studies confirmed that the matrix would remain stable for prolonged time periods (>42 days in culture medium). This degradation profile is typical for ester-containing hydrogels mediated by hydrolytic cleavage of the ester bonds within PDLLA [20,32]. In the presence of cells, degradation is enhanced by the activity of secreted matrix metalloproteinases (MMPs) [31,33]. Collagen deposition and collagen fibre formation increased under dynamic stimulation conditions, indicating improved matrix turnover under dynamic stimulation. Overall, the longevity of the samples suggests that GelMA/P(LA-EG-LA)-bMA hydrogels retain sufficient structural integrity for longer durations than that required to support tendon differentiation of encapsulated hBMSCs, allowing ample time for the deposition of new matrix. Importantly, the gels were also able to withstand the forces applied using the Flexcell system for dynamic mechanical stimulation. Together, these properties are sufficient for modelling tenogenic differentiation *in vitro*.

When encapsulating cells within a 3D hydrogel, factors including the residual presence of the hydrogel precursors and degradation products, and culture setup can all have a negative impact on cell viability. At 7 days post-encapsulation, cell viability was 86 ​± ​1% showing above sufficient viability according to ISO standard 10993-5. Furthermore, cell proliferation studies showed Ki67-expression in cells derived from all donors confirming hBMSC proliferation within the hydrogel while F-actin filament staining showed cells being able to spread. These data show the biocompatibility of the GelMA/P(LA-EG-LA)-bMA hydrogels for 3D hBMSC culture.

Assessment of the induction of tenogenic gene expression showed substantial increases in *SCX* and *COL1A1* expression*,* but relatively little change in *TNC* and *COL3A1*. Interestingly, expression increased in all the elongated hydrogel constructs compared to the hydrogel droplet, although with no further increase under either static or dynamic physical stimulation. This finding suggests that a simple change in hydrogel geometry to an elongated shape can positively influence tenogenesis, likely due to the more aligned cell phenotype in the former as was observed in H&E staining. Such elongated cells were evident in all of the samples, oriented with the periphery of the elongated hydrogel structures. This may be beneficial because cell alignment has been shown to improve tenogenic differentiation [[34], [35], [36]]. TGF-β3 provision substantially increased the expression of *SCX* and *COL1A1* at both 5 ​ng/ml and 10 ​ng/ml concentrations. TGF-β3 alone has previously shown to increase SCX expression and in combination with alignment also increased COL1A1 expression [14,37,38]. In the mouse, SCX is expressed by tenogenic progenitor cells (TPCs) from embryonic day (E) 9.5 into adulthood [39,40]. Muscle-induced mechanical stimulation starts at E14 [41], which suggests mechanical stimulation is needed for proper tenogenic differentiation. Ectopic TGF-β3 supplied for 5 ​day ​at 4, 20, and 100 ​ng/ml was shown to upregulate SCX at protein level but not at gene level [42]. We show here that after 7 days of supplementation, expression at the gene level can be observed.

We noted no strong benefit in combining biochemical and physical stimulation upon gene expression levels, or between static and dynamic stimulation. This was unexpected given that previous reports have suggested that *SCX* expression is induced by TGF-β signalling and further modulated by mechanical loading [43]. For example, cyclic mechanical loading at 1 ​Hz, 0–6% sinusoidal wave strain for 18 ​h showed increased SCX expression in BMSCs [42]. Our observations could be due to the high swelling ratio of the hydrogel. The hydrogel was anchored prior to swelling which may have decreased the effect of the dynamic mechanical stimulation, considering the change in G′ from 1930 ​± ​230 ​Pa (pre-swelling) to 200 ​± ​40 ​Pa (post-swelling) and the high swelling ratio (16.5 ​± ​0.4). The elastic modulus of embryonic tendons is typically higher than these values, ranging between 9 and 31 ​kPa [[44], [45], [46]]. This lower G′ of the GelMA/P(LA-EG-LA)-bMA hydrogel is a limitation of this study in terms of transmitting the mechanical stimulation from the Flexcell to the construct. However, matrix deposition following cell culture is expected to increase the G’, and hydrogels with a low modulus have generally proven more effective in supporting matrix deposition and new tissue formation [47]. For this reason, changing the composition of the hydrogel to provide a higher modulus was not tested in this study. The maximum strain for tendons *in vivo* is 4%, with higher strain levels potentially causing tissue damage [48]. Based on this and a similar protocol used in a custom-made bioreactor [8], we selected 3% elongation at 0.33 ​Hz for 1 ​h/day as our stimulation regimen. The model developed in this study therefore gives insight into the key players regulating tenogenic differentiation of hBMSCs that may be further pursued *in vivo*. Due to the high swelling ratio of the hydrogel scaffold, it is hypothesized that an alternative dynamic stimulation regimen with increased strain could more effectively increase *SCX* expression, as for example seen in a study using collagen type I gels, where dynamic stimulation was applied using the Flexcell system with 1% elongation, at 0.33 ​Hz for 30 ​min/day [7]. Similarly, dynamic stimulation applied to BMSCs in a custom-made bioreactor increased *SCX* expression by five-fold [49]. Increasing the level of strain applied during dynamic stimulation might therefore improve *SCX* expression within the GelMA/P(LA-EG-LA)-bMA hydrogel. TGF-β3 has previously been shown to increase *COL1A1*, *COL3A1* and *TNC* expression at 10 ​ng/ml, the highest concentration used in this study [13,50]. We observed similar effects on *COL1A1*, but not *COL3A1* or *TNC* transcription. However, the regulation of *TNC* expression throughout tenogenesis is highly dynamic and so the differences in these results could also be due to analysis carried out at different time points.

Although mechanical stimulation did not increase the expression of tenogenic genes, it did affect the deposition of matrix and organisation of collagen within the tenogenic constructs. Constructs stimulated with dynamic mechanical forces showed distinctively different Alcian Blue staining patterns, although not significantly following quantitation. Birefringence microscopy did show a significant increase in collagen fibre formation for DS-5 and DS-10 in Donor 1, with Donor 2 and 3 also showing an increase but at too high variability to be significant. Donor 2 and 3 showed more variability, but overall ectopic supplementation with 5 ​ng/ml TGF-β3 improved collagen fibre formation. Our observations showed that TGF-β3 generally had a positive effect on matrix formation, with the strongest effects observed in DS samples. We observed PG content non-significantly increased most in response to TGF-β3 in DS samples, but not those under static stimulation. The content of PGs in engineered tendon is important due to their role in the viscoelastic properties of tendons by trapping water [51]. The amount and composition of PGs in the tendon are also a key indicator of tendon health. Although high PG levels are often linked to tendinopathies in adults [52], embryonic chick tendons have high GAG deposits, indicating high PG content during tendon development [45]. Overall, this supports that the increased GAG deposition, induced by dynamic stimulation is an indicator of an improved tenogenic response. A seven-day culture period was chosen to be comparable to other tenogenic differentiation studies and is expected to show the early stages of tenogenic differentiation. However, it can also be seen as a limitation of this study, considering that longer cell culture times would give further insight into the process of matrix deposition in the course of tenogenesis. To improve matrix deposition, future work may seek to incorporate small molecules and/or growth factors such as BMP4 [53], to improve differentiation. Further studies using a two-step differentiation protocol consisting of an initiation phase by biochemical stimulation followed by mechanical and biochemical stimulation would be of interest as this has previously been shown to enhance tenogenic differentiation of hBMSCs [14].

The strength of tendon tissue is primarily imparted by the hierarchically structured collagen fibres and collagen fibre formation is thus an important feature in tendon development. In terms of collagen deposition and organisation, the combined results from the Picrosirius red and collagen-I staining, as well as the fibrous nature shown by birefringence microscopy, suggest a positive effect of dynamic stimulation in combination with TGF-β3 supplementation. Our data also showed differences in the amount of collagen deposited in different areas of the samples, likely influenced by the location of the cells which were depositing this matrix. This is evidenced by the greater collagen deposition at the periphery of the construct, where there was a high density of elongated cells, and in areas around the cavities in the gel in which the cells were typically resident (Fig. 2C; 5C; Supplementary Figs. 13–16).

## 5 Conclusion

This study systematically investigated the interplay of TGF-β3 and mechanical stimulation on the tenogenic differentiation of hBMSCs in a 3D GelMA/P(LA-EG-LA)-bMA hydrogel. The hydrogel scaffold was confirmed to support cell activity and had the required mechanical strength to support physical stimulation. Overall, samples supplemented with 5 ​ng/ml TGF-β3 under intermittent cyclic uniaxial strain (3% strain; 0.33 ​Hz; 1 ​h/day) showed the most promising result for tenogenic differentiation of hBMSCs, with the greatest induction of SCX and COL1A1 expression and the highest increase in matrix deposition and organisation. Our findings show that TGF-β3 provision is effective to upregulate the tenogenic genes *SCX* and *COL1A1* but that physical stimulation is necessary for improved matrix deposition, with a higher collagen fibre formation being observed for dynamically stimulated samples compared to static stimulation. Due to the interplay between mechanical stimulation and TGF-β3, matrix deposition is higher in 5 ​ng/ml TGF-β3 supplemented dynamic gels than 10 ​ng/ml TGF-β3. The knowledge gained from this study shows the need for both biochemical and dynamic biophysical stimulation for tenogenic differentiation of hBMSCs, and that dynamic stimulation balanced with intermediate TGF-β3 concentration levels has the greatest impact on gene expression and ECM deposition. This information will contribute to the design of an optimised tenogenic differentiation protocol for tendon tissue engineering, with further improvements likely to be made by further comparison of different dynamic loading regimens. Furthermore, our results on the positive effects on hBMSC tenogenic differentiation after just 7 days of culture are highly encouraging and provide a promising insight into the future potential to generate tendon tissue via MSC-based tissue engineering.

## Authorship

All persons who meet authorship criteria are listed as authors, and all authors certify that they have participated sufficiently in the work to take public responsibility for the content, including participation in the concept, design, analysis, writing, or revision of the manuscript. Each author certifies that this material or part thereof has not been published in another journal, that it is not currently submitted elsewhere, and that it will not be submitted elsewhere until a final decision regarding publication of the manuscript in Journal of Orthopaedic Translation has been made.

Conception and design of study: Jessica E. Frith, Rocky S. Tuan. Acquisition of data: Ilze Donderwinkel. Analysis and/or interpretation of data: Ilze Donderwinkel. Drafting the manuscript: Ilze Donderwinkel. Revising the manuscript critically for important intellectual content: Jessica E. Frith, Rocky S. Tuan, Neil R. Cameron. Approval of the version of the manuscript to be published : Ilze Donderwinkel, Rocky S. Tuan, Neil R. Cameron, Jessica E. Frith.

## Figures and Tables

**figs1:**
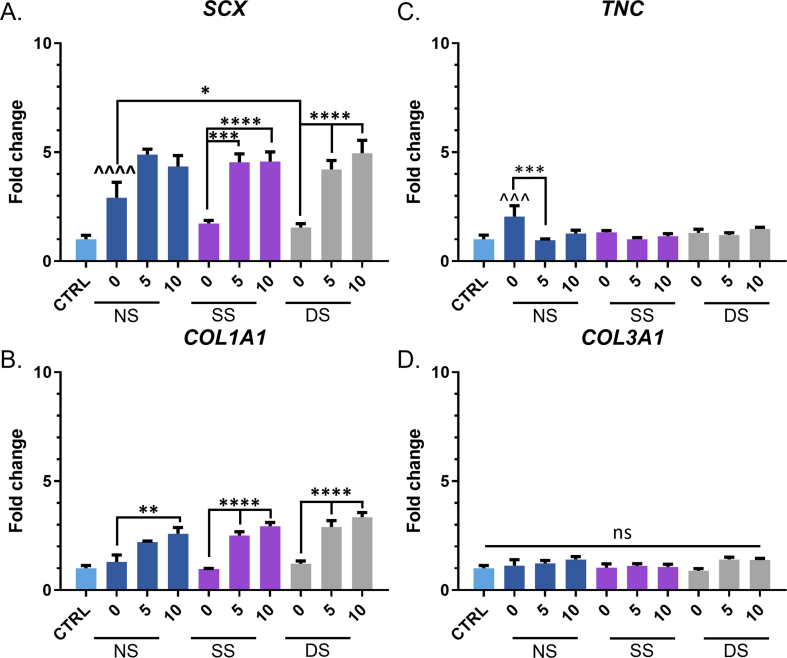


**figs2:**
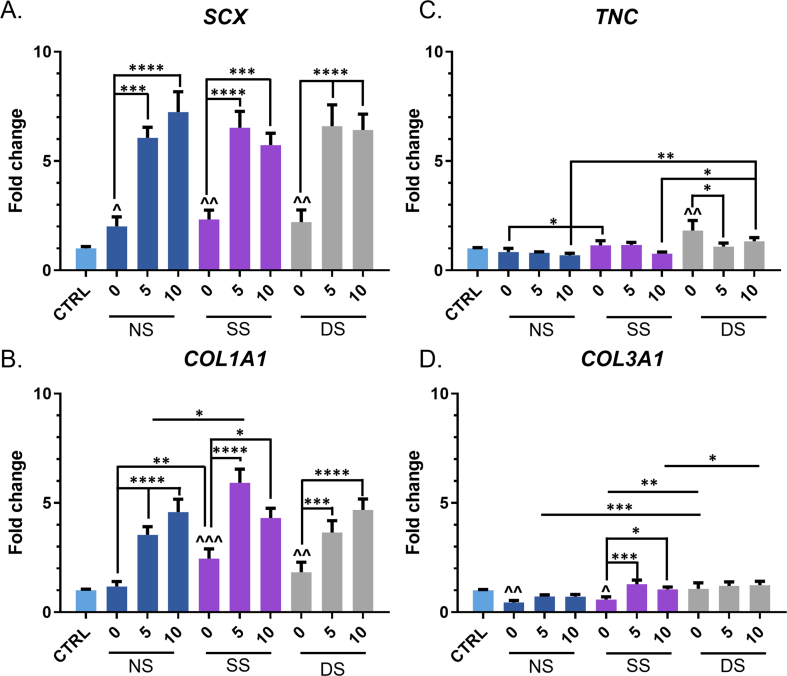


**figs3:**