# Supplementary material for: A systematic investigation of the effects of TGF-β3 and mechanical stimulation on tenogenic differentiation of mesenchymal stromal cells in a poly(ethylene glycol)/gelatin-based hydrogel
Source: J Orthop Translat. 2023 Oct 21;43:1–13. doi: 10.1016/j.jot.2023.09.006 (PMC10622696; doi:10.1016/j.jot.2023.09.006)
Supplement: Multimedia component 1 [file mmc1.pdf]

Supporting information

A systematic investigation of the effects of TGF-β3 and mechanical stimulation on tenogenic differentiation in a poly(ethylene glycol)/gelatin-based hydrogel

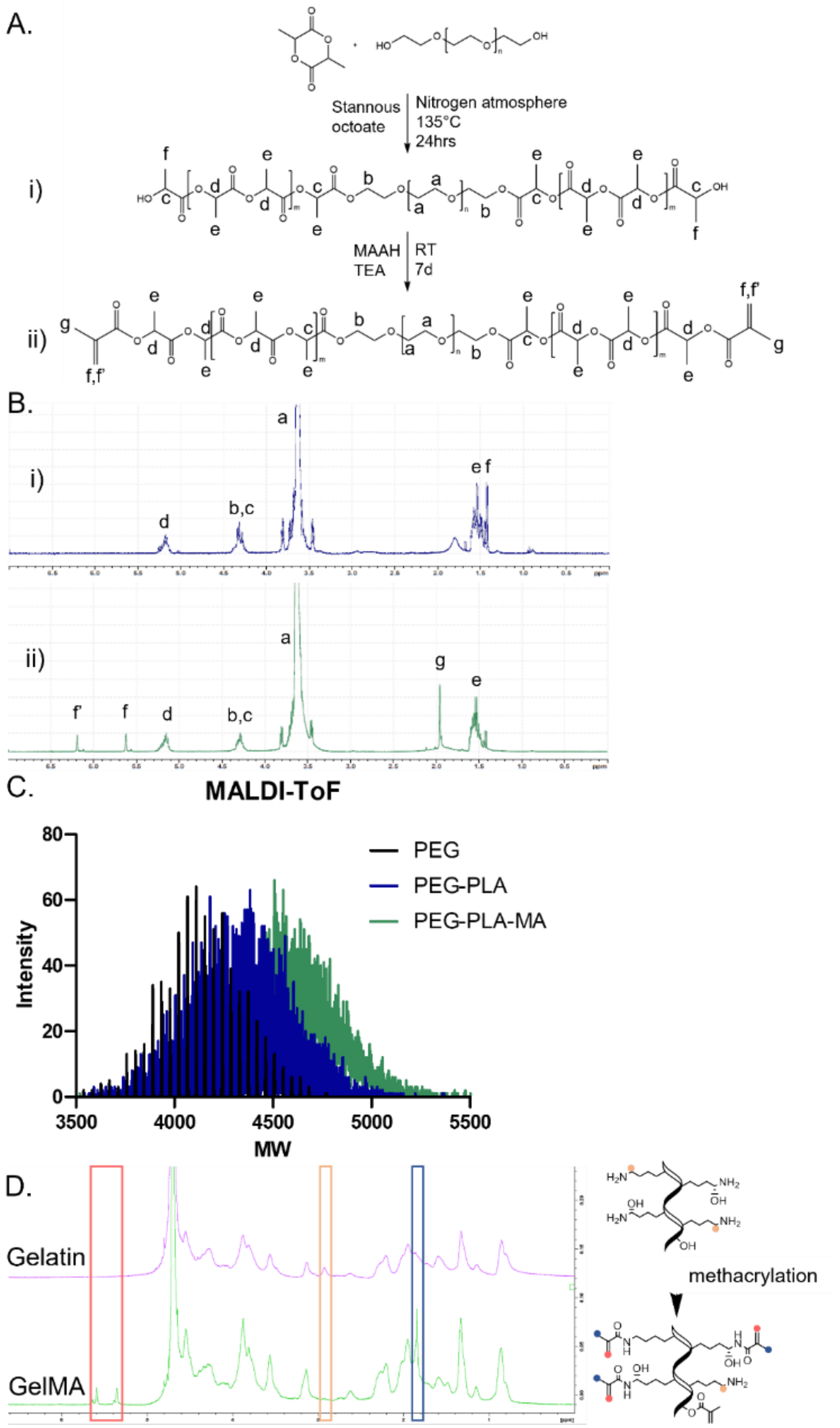

Supplementary Figure 1. Polymer synthesis. (A) Reaction mechanism of ring opening polymerisation of D,L-lactide initiated by PEG and methacrylation. (B) ^{1}H-NMR spectra of P(LA-EG-LA) (Bi)) and P(LA-EG-LA)-bMA (Bii)). (C) MALDI of PEG, P(LA-EG-LA) and P(LA-EG-LA)-bMA. (D) ^{1}H-NMR spectra of gelatin

methacrylation showing emergence of the methacrylate methyl proton peak (δ1.86 ppm, blue) and methacrylate methylene proton peaks (δ5.37 ppm and δ.560 ppm, red), and decrease of the lysine methylene proton peak (δ2.84-2.94 ppm, orange).

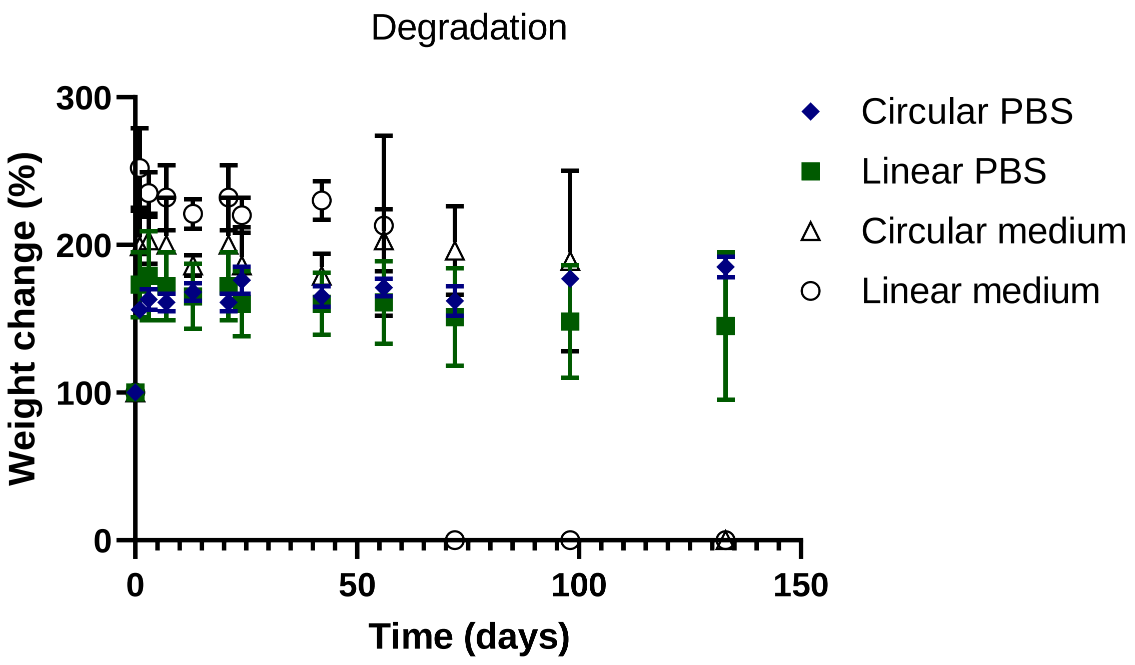

Supplementary Figure 2. Degradation of circular and linear hydrogel constructs in DPBS or cell culture medium. Graph is shown as mean ± SD, N=3.

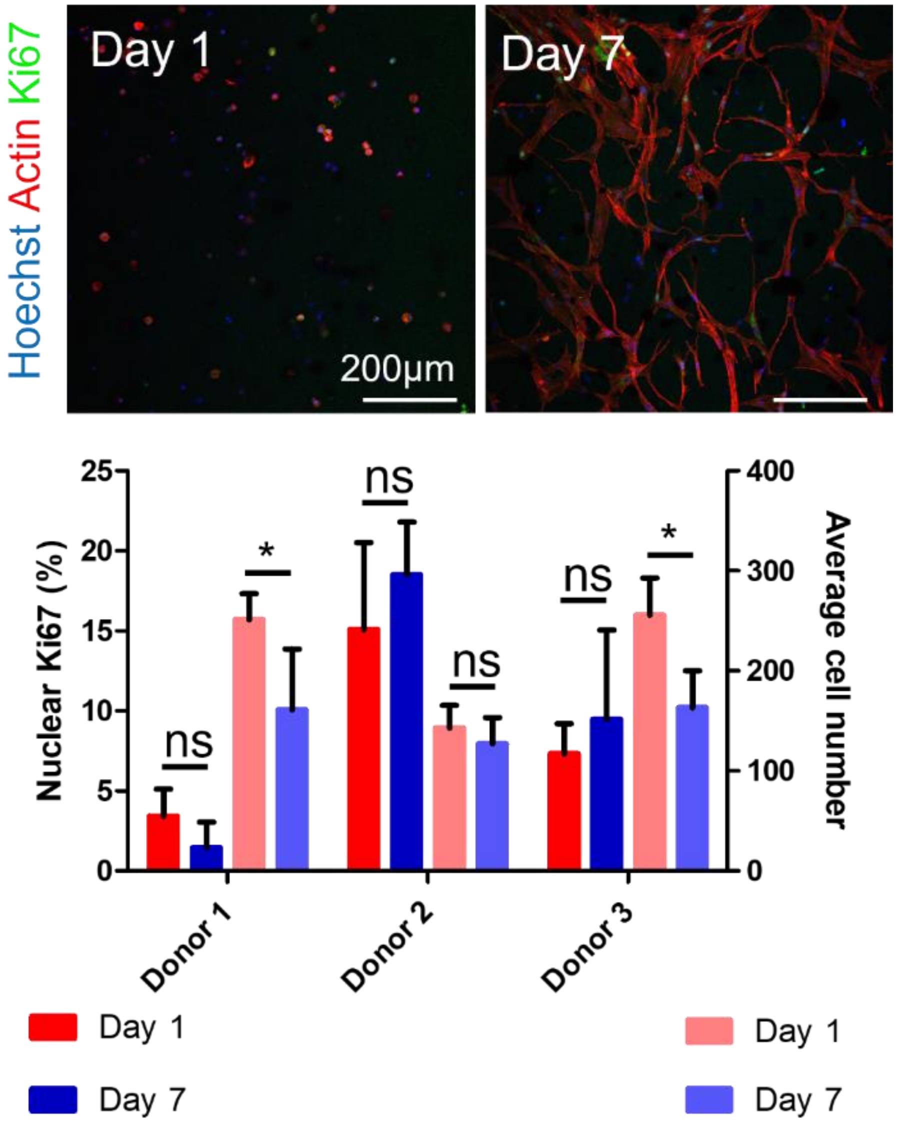

Supplementary Figure 3. Cell spreading by F-actin filament staining, including cell proliferation by Ki67 staining for day 1 and 7. Percentage of nuclear Ki67 positive cells and average cell number (cells/FOV (512x512)) after 1 day and 7 days of hBMSC encapsulation. Graphs are shown as mean ± SD, N = 3; ns, p>0.05; *, p≤0.05; **, p≤0.01; ***, p≤0.001.

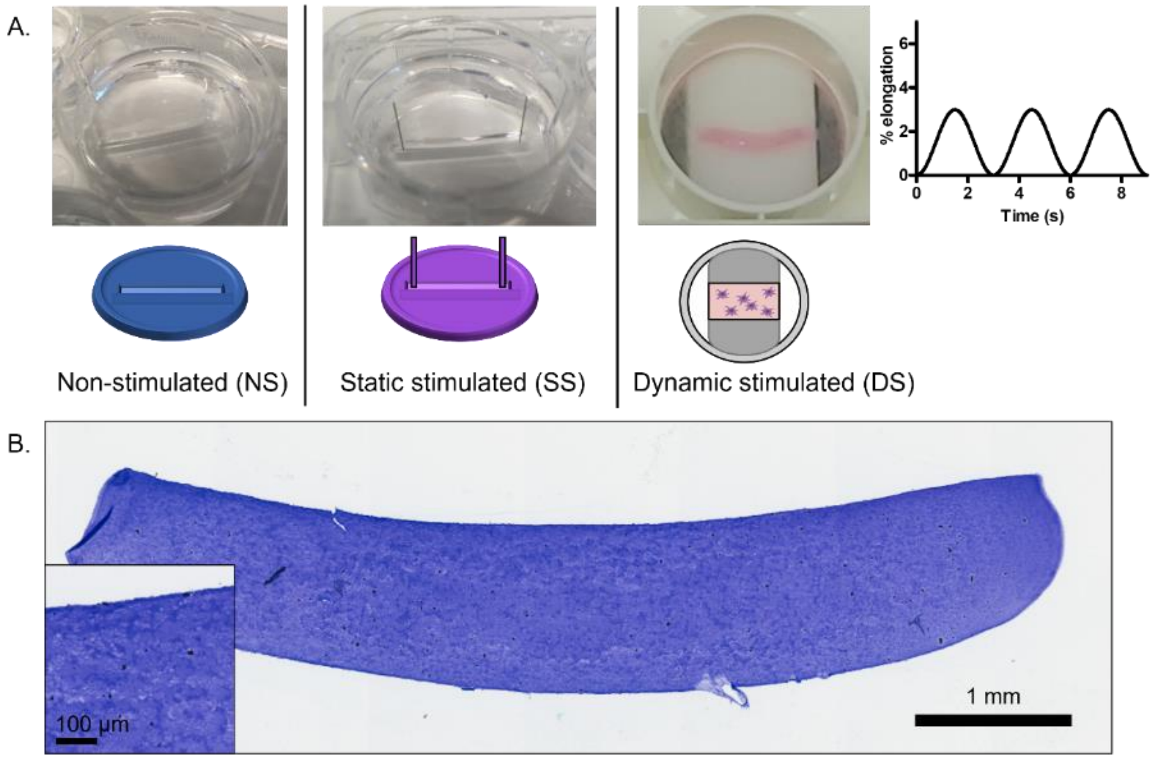

Supplementary Figure 4. Design for mechanical stimulation of hydrogels. (A) Preparation of static setup by inverted PDMS mould prepared using the Flexcell system, followed by non-stimulated PDMS trough (NS) and static stimulation using minutien pins (SS). Dynamic stimulation using intermittent cyclic uniaxial strain was achieved using the Flexcell FX-4000T Tension system at 3% elongation, 0.33 Hz for 1 h/day (DS). (B) Toluidine blue stained histological sections show no degradative effect due to mechanically stimulation to the hydrogel constructs. Scale bar = 1 mm (full); 100 µm (inset).

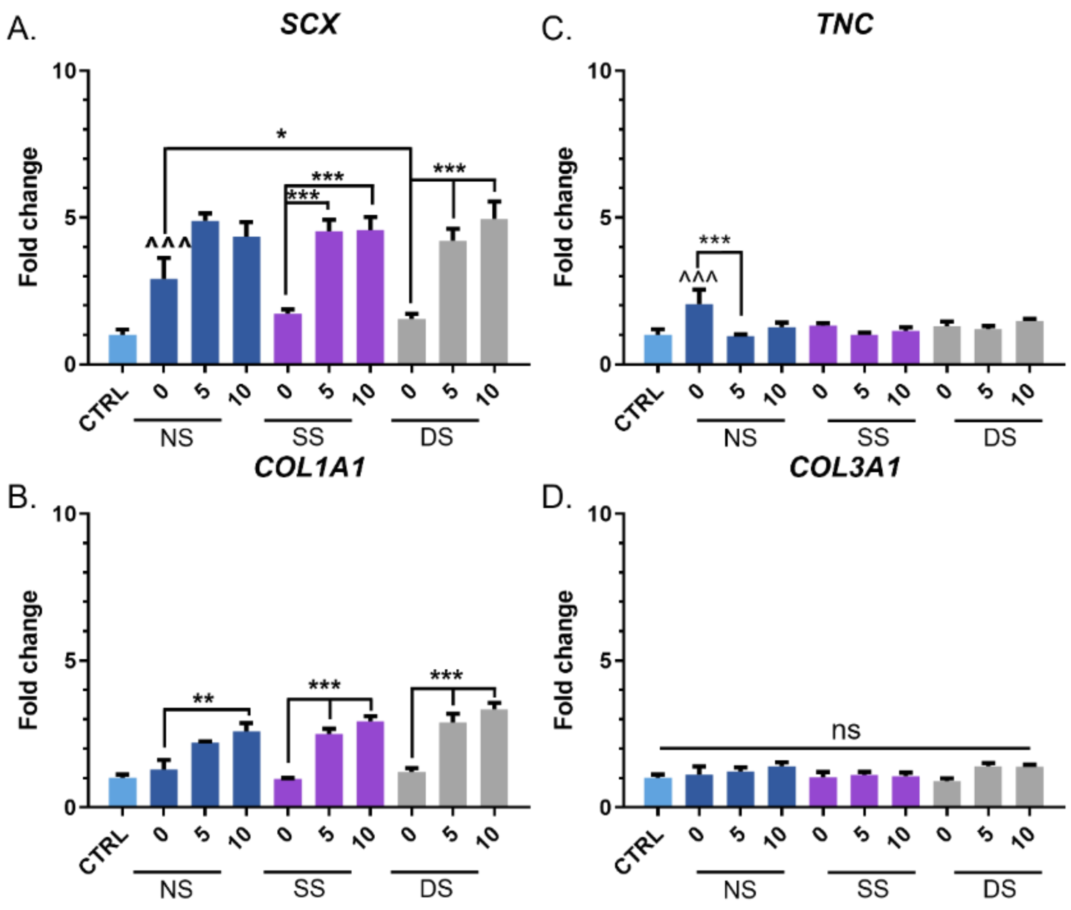

Supplementary Figure 5. Tenogenic gene expression for **Donor 1** of (A) scleraxis (SCX), (B) collagen type I (COL1A1), (C) tenascin c (TNC), and (D) collagen type III (COL3A1) in hBMSC-hydrogel constructs as a function of TGF-β3 treatment dose and mechanical stimulation after culture day 7. Analysis was performed on linear hydrogel encapsulated hBMSCs after 7 days of cull culture without stimulation (NS), or under static (SS) or dynamic (DS) mechanical stimulation, in absence (0) or presence of 5 ng/ml (5) or 10 ng/ml (10) TGF-β3. Encapsulated hBMSCs in a bulk hydrogel (droplet) cultured for 7 days were used as a control (CTRL). Graphs are shown as mean ± SD, N = 3; ns, p>0.05; *, p≤0.05; **, p≤0.01; ***, p≤0.001. Significant differences to the CTRL are shown in ^.

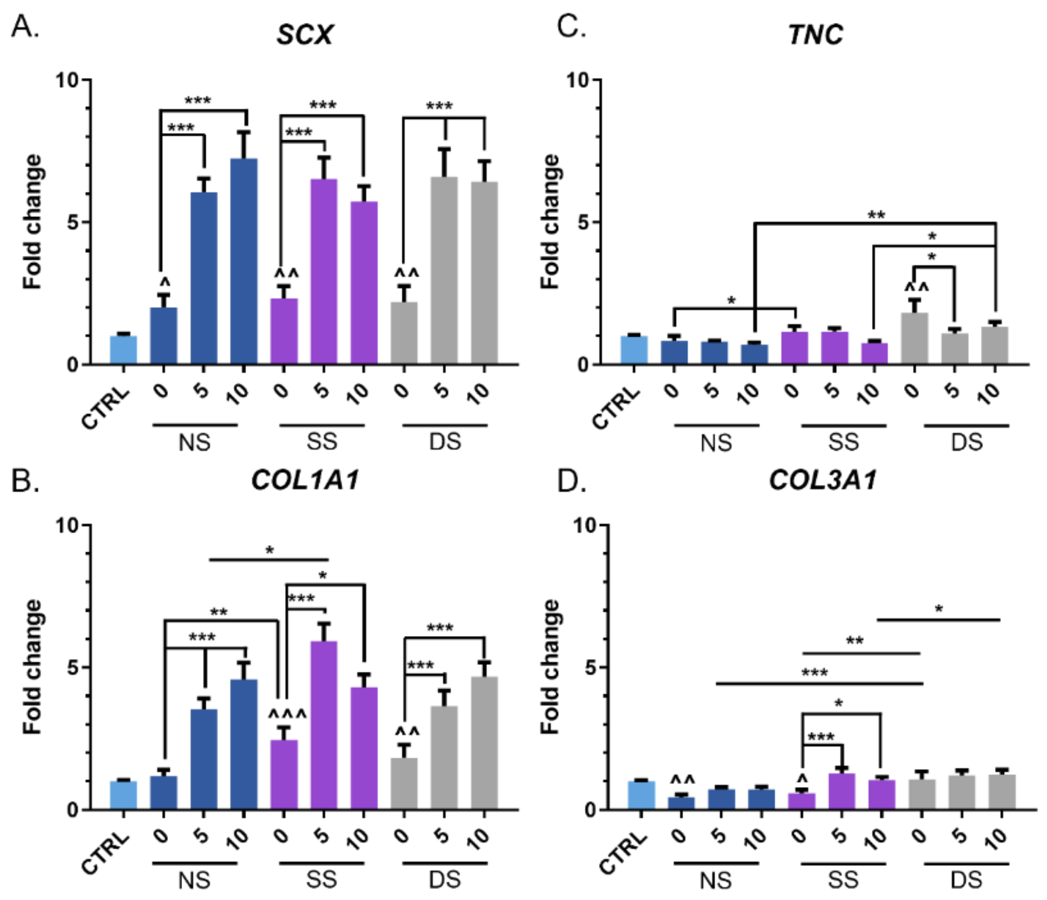

Supplementary Figure 6. Tenogenic gene expression for **Donor 2** of (A) scleraxis (SCX), (B) collagen type I (COL1A1), (C) tenascin c (TNC), and (D) collagen type III (COL3A1) in hBMSC-hydrogel constructs as a function of TGF-β3 treatment dose and mechanical stimulation after culture day 7. Analysis was performed on linear hydrogel encapsulated hBMSCs after 7 days of cull culture without stimulation (NS), or under static (SS) or dynamic (DS) mechanical stimulation, in absence (0) or presence of 5 ng/ml (5) or 10 ng/ml (10) TGF-β3. Encapsulated hBMSCs in a bulk hydrogel (droplet) cultured for 7 days were used as a control (CTRL). Graphs are shown as mean ± SD, N = 3; ns, p>0.05; *, p≤0.05; **, p≤0.01; ***, p≤0.001. Significant differences to the CTRL are shown in ^.

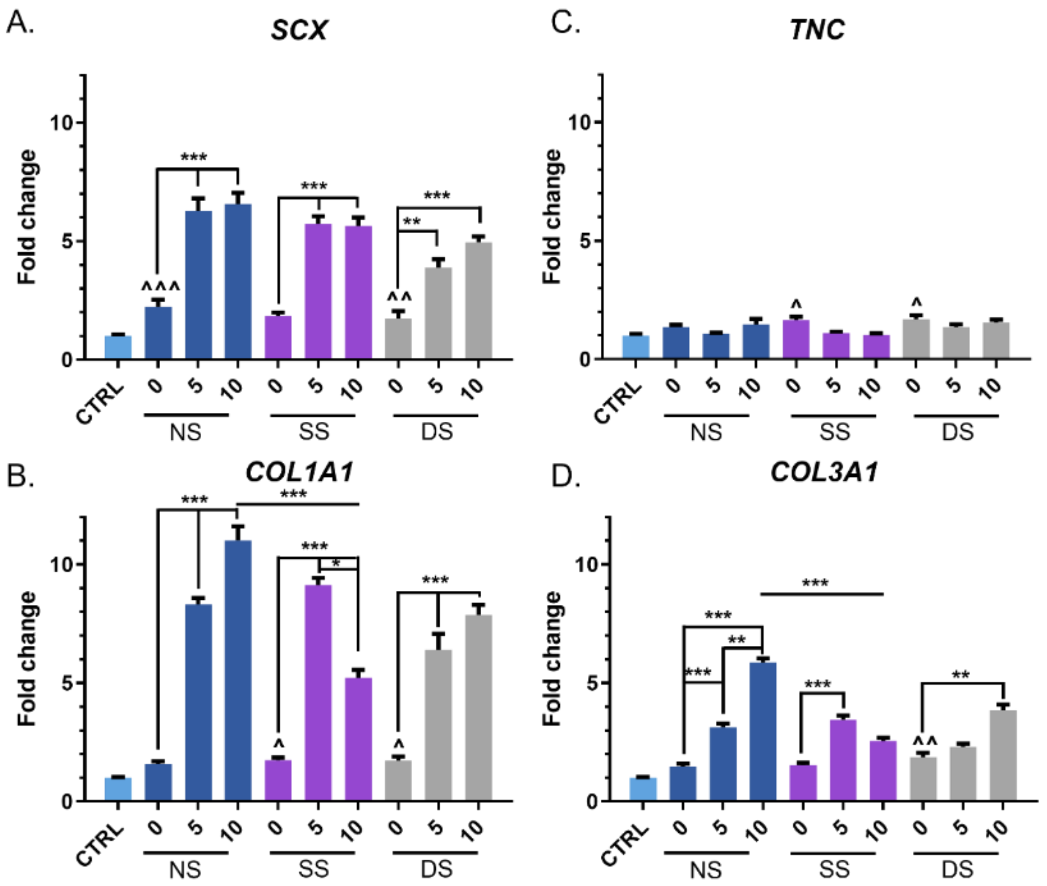

Supplementary Figure 7. Tenogenic gene expression for **Donor 3** of (A) scleraxis (SCX), (B) collagen type I (COL1A1), (C) tenascin c (TNC), and (D) collagen type III (COL3A1) in hBMSC-hydrogel constructs as a function of TGF-β3 treatment dose and mechanical stimulation after culture day 7. Analysis was performed on linear hydrogel encapsulated hBMSCs after 7 days of cull culture without stimulation (NS), or under static (SS) or dynamic (DS) mechanical stimulation, in absence (0) or presence of 5 ng/ml (5) or 10 ng/ml (10) TGF-β3. Encapsulated hBMSCs in a bulk hydrogel (droplet) cultured for 7 days were used as a control (CTRL). Graphs are shown as mean ± SD, N = 3; ns, p>0.05; *, p≤0.05; **, p≤0.01; ***, p≤0.001. Significant differences to the CTRL are shown in ^.

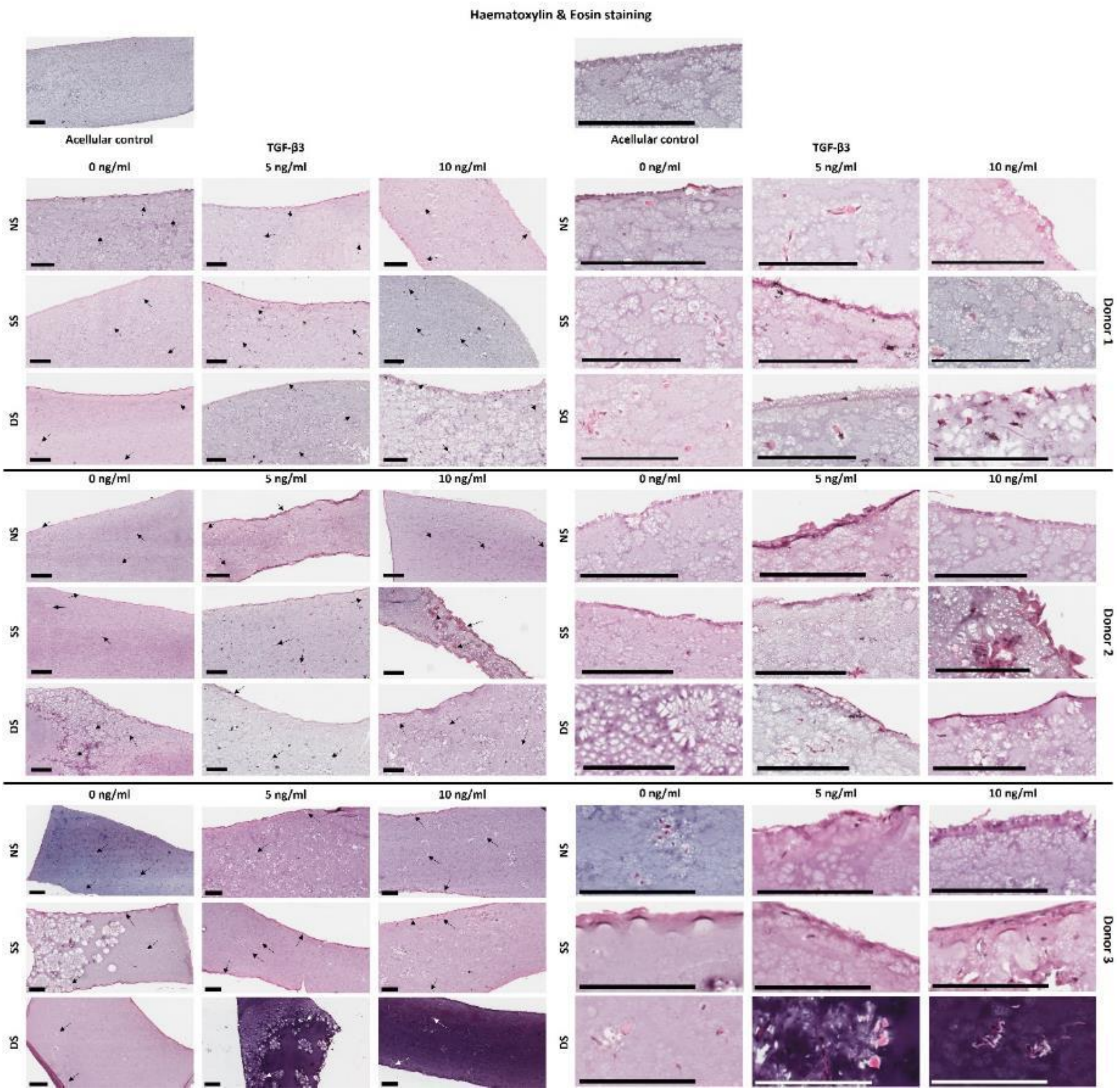

Supplementary Figure 8. Histological analysis (Haematoxylin & Eosin staining) of the effects of TGF-β3 treatment dose and mechanical stimulation on hBMSC-hydrogel construct on culture day 7. Cell nuclei are indicated by black arrows. Scale bar = 200 µm. Images show representative sections of three technical replicates.

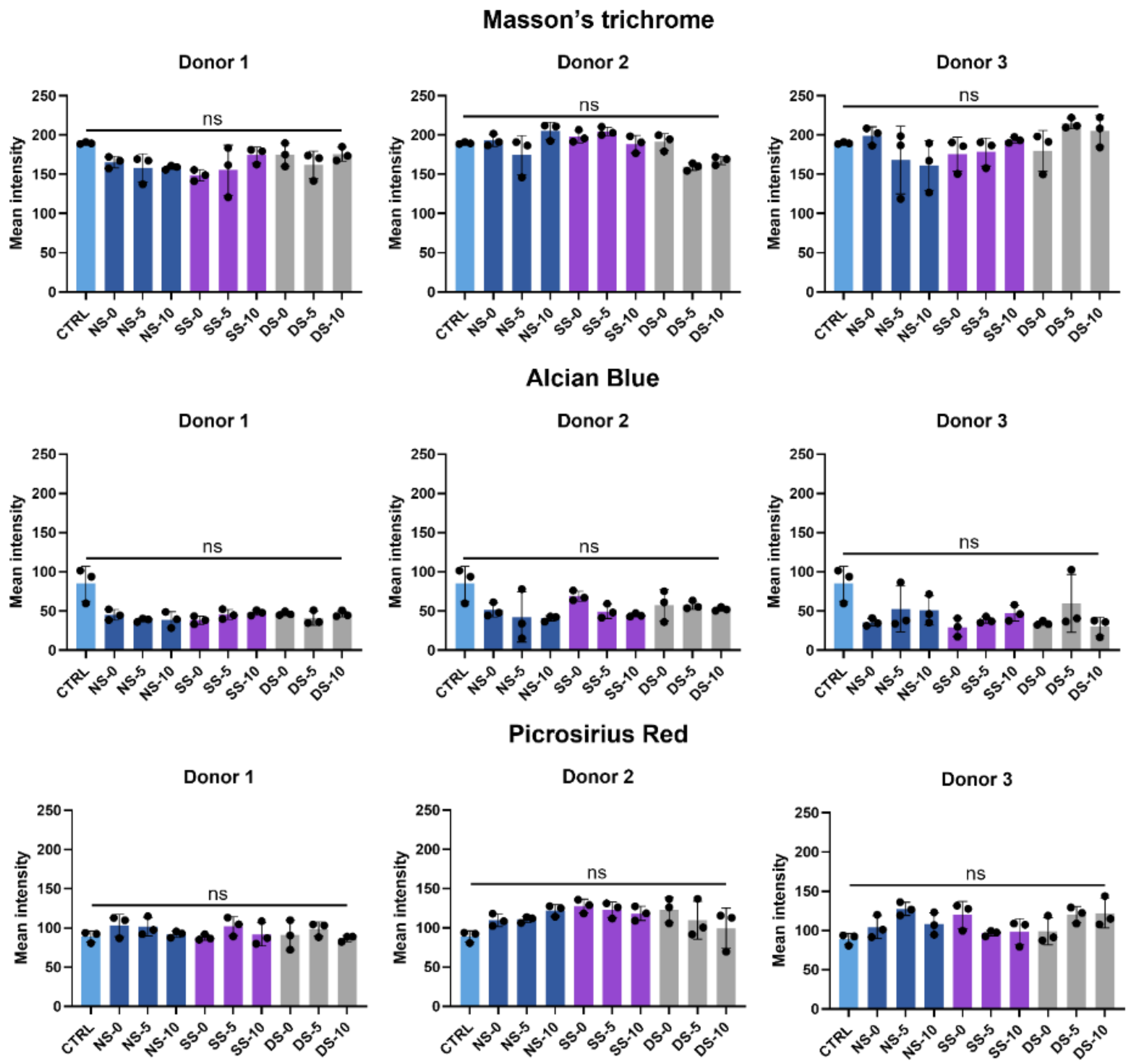

Supplementary Figure 9. Comparison of histological staining intensity of three technical replicates relative to the acellular control for Masson's trichrome, Alcian Blue and Picrosirius Red. Graphs are shown as mean ± SD, N = 3; ns, p>0.05; *, p≤0.05; **, p≤0.01; ***, p≤0.001.

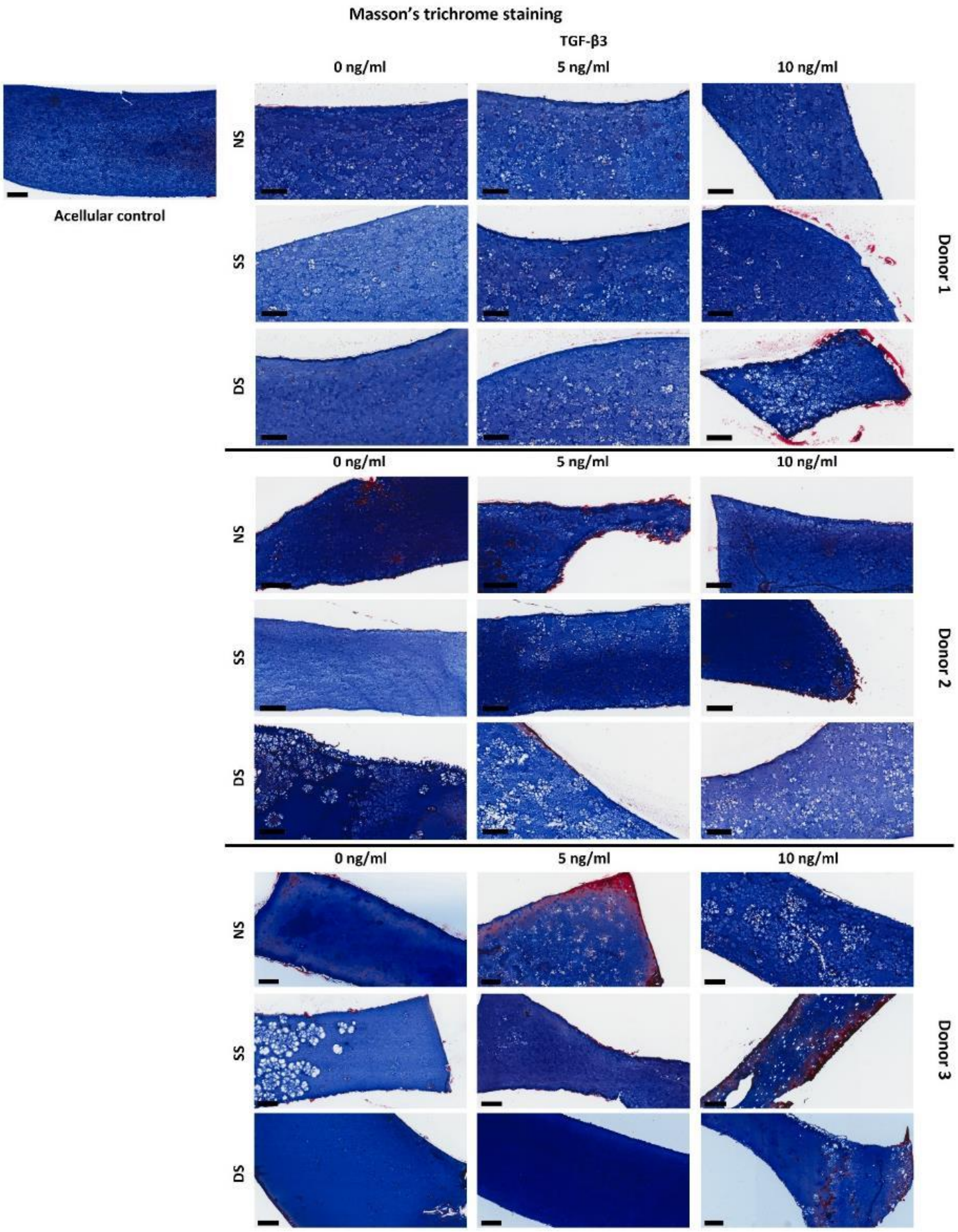

Supplementary Figure 10. Histological analysis by Masson's trichrome staining of the effects of TGF-β3 treatment dose and mechanical stimulation on hBMSC-hydrogel construct on culture day 7. Scale bar = 200 µm. Images show representative sections of three technical replicates.

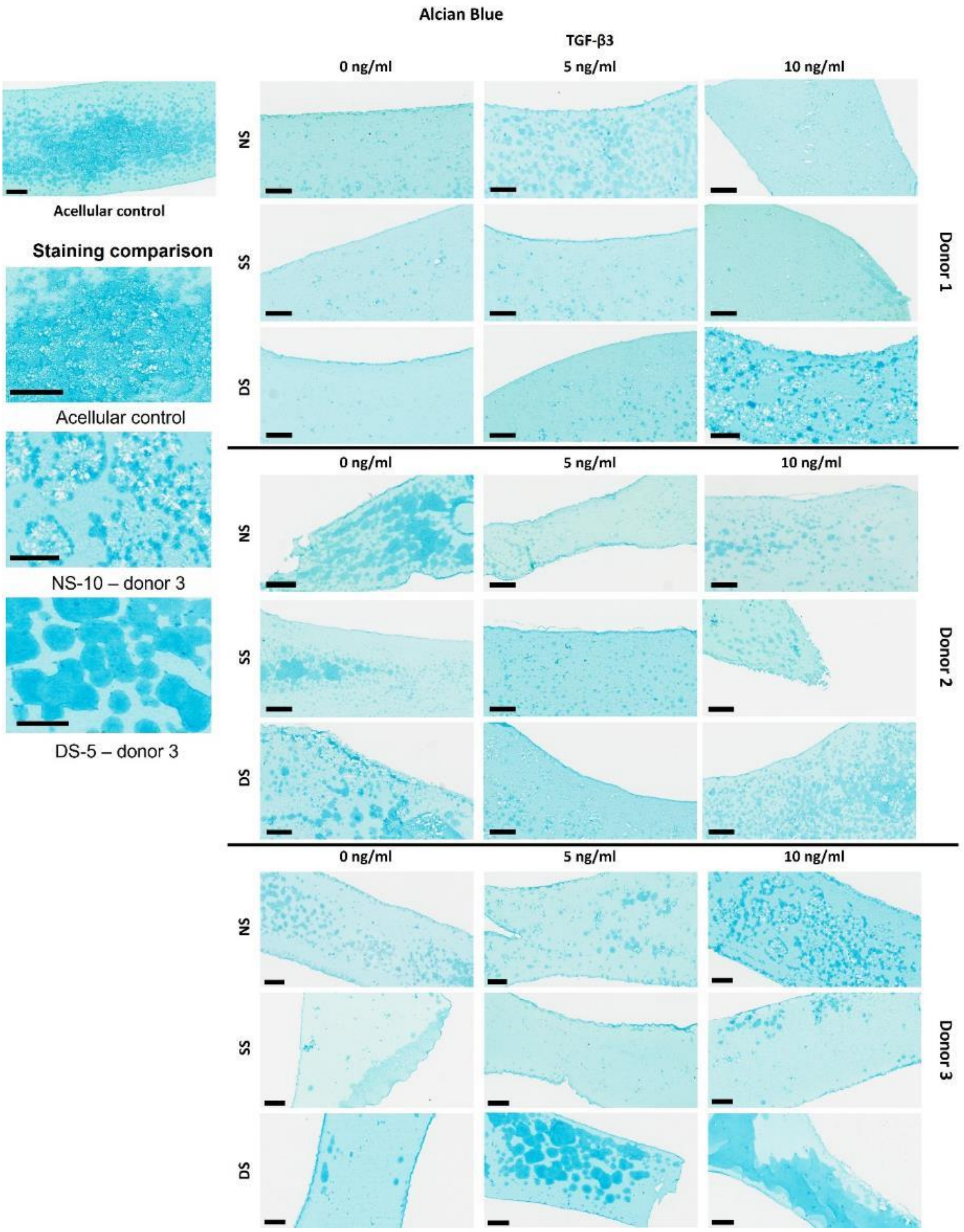

Supplementary Figure 11. Histological analysis by Alcian blue staining of the effects of TGF-β3 treatment dose and mechanical stimulation on hBMSC-hydrogel construct on culture day 7. Scale bar = 200 µm. Images show representative sections of three technical replicates.

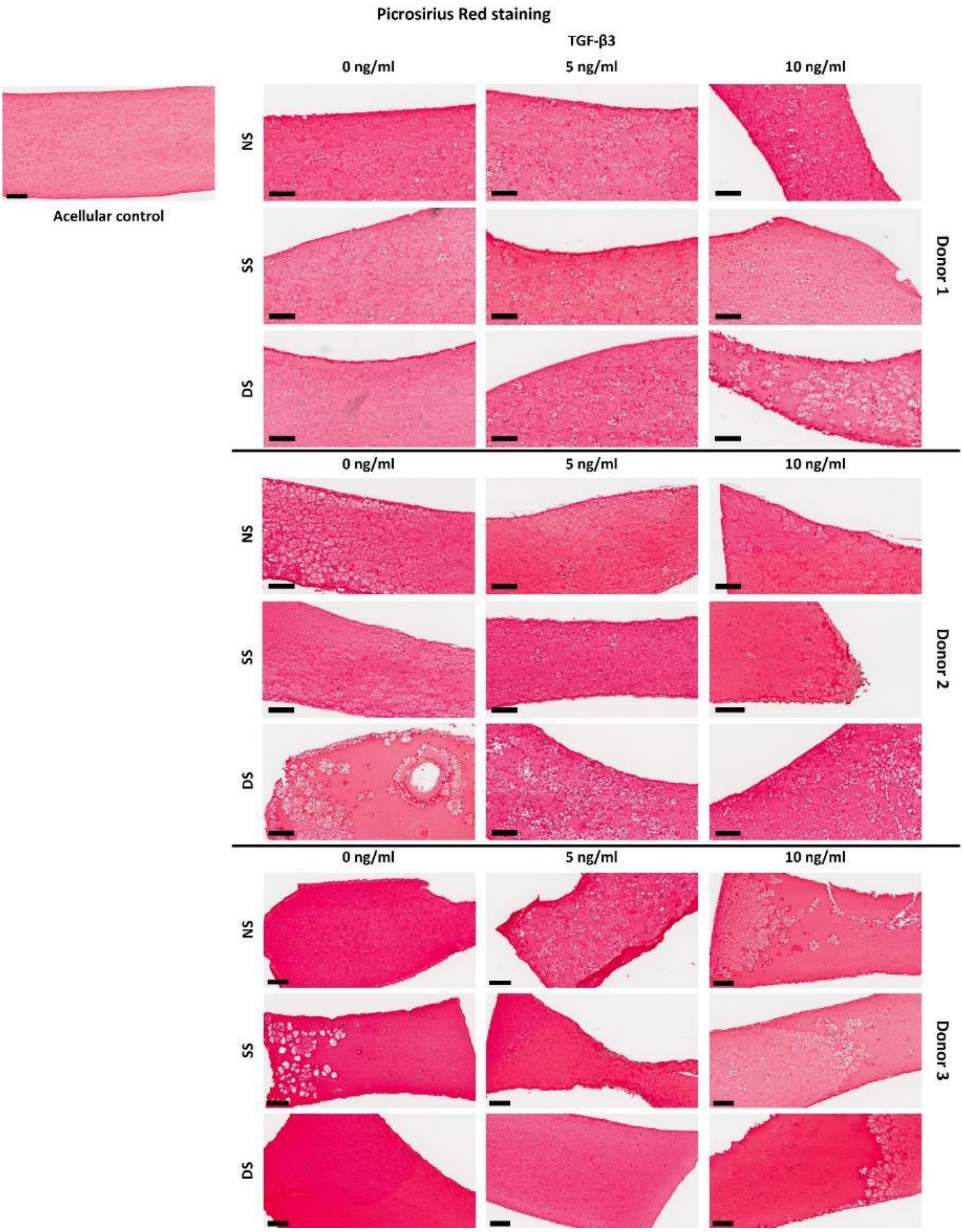

Supplementary Figure 12. Histological analysis by Picrosirius red staining of the effects of TGF-β3 treatment dose and mechanical stimulation on hBMSC-hydrogel construct on culture day 7. Scale bar = 200 µm. Images show representative sections of three technical replicates.

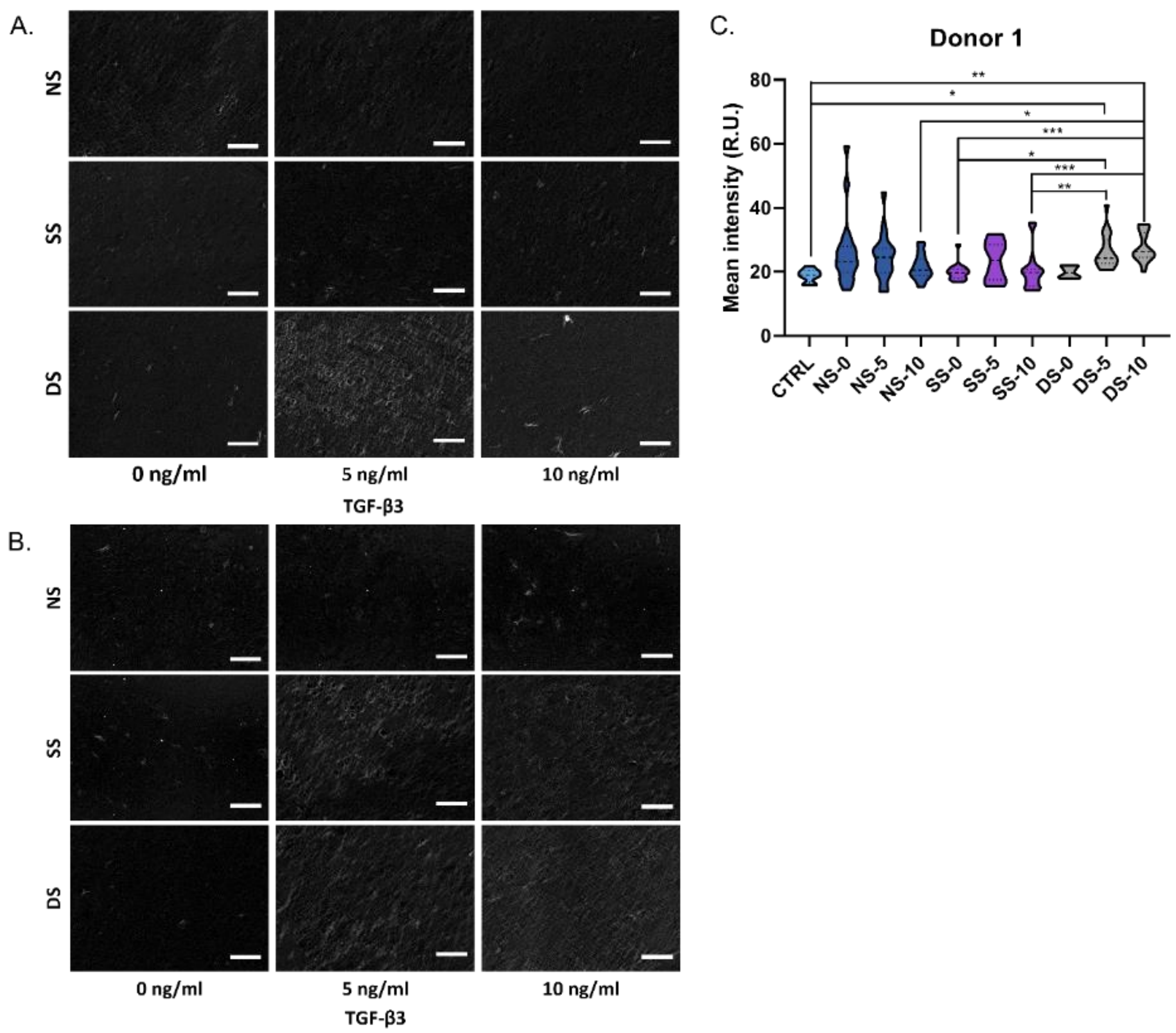

Supplementary Figure 13. Birefringence microscopy images of picrosirius red stained sections near the surface (A) and centre (B) of the hydrogel for Donor 1 (N=3). Collagen fibres appear white against the grey background. (C) Quantitation of birefringence microscopy image intensity of triplicate measures of biological and technical replicates. Scale bar = 100 µm. Graphs are shown truncated violin plots, N = 3; ns, p>0.05; *, p≤0.05; **, p≤0.01; ***, p≤0.001.

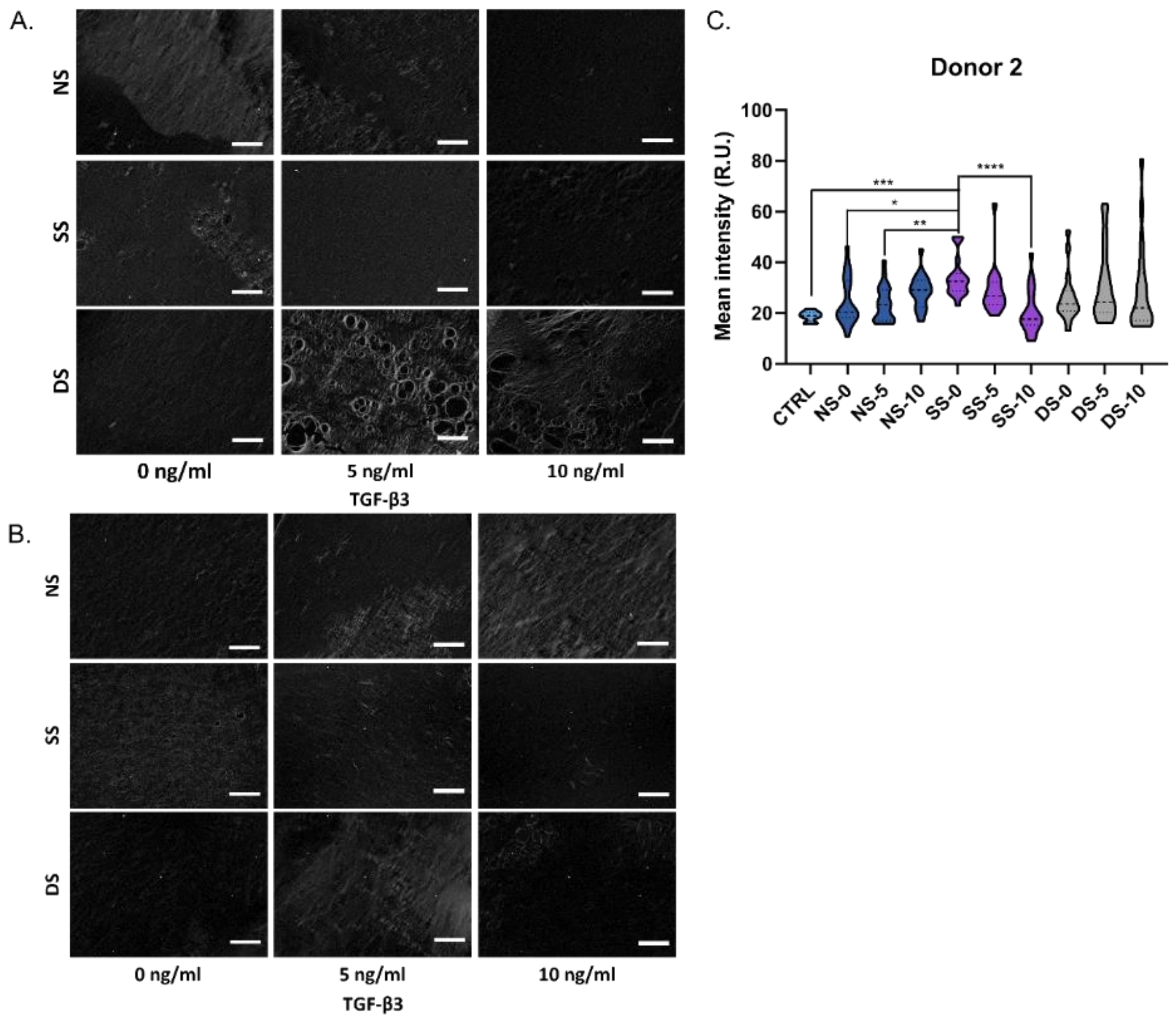

Supplementary Figure 14. Birefringence microscopy images of picrosirius red stained sections near the surface (A) and centre (B) of the hydrogel for Donor 2 (N=3). Collagen fibres appear white against the grey background. (C) Quantitation of birefringence microscopy image intensity of triplicate measures of biological and technical replicates. Scale bar = 100 µm. Graphs are shown truncated violin plots, N = 3; ns, p>0.05; *, p≤0.05; **, p≤0.01; ***, p≤0.001.

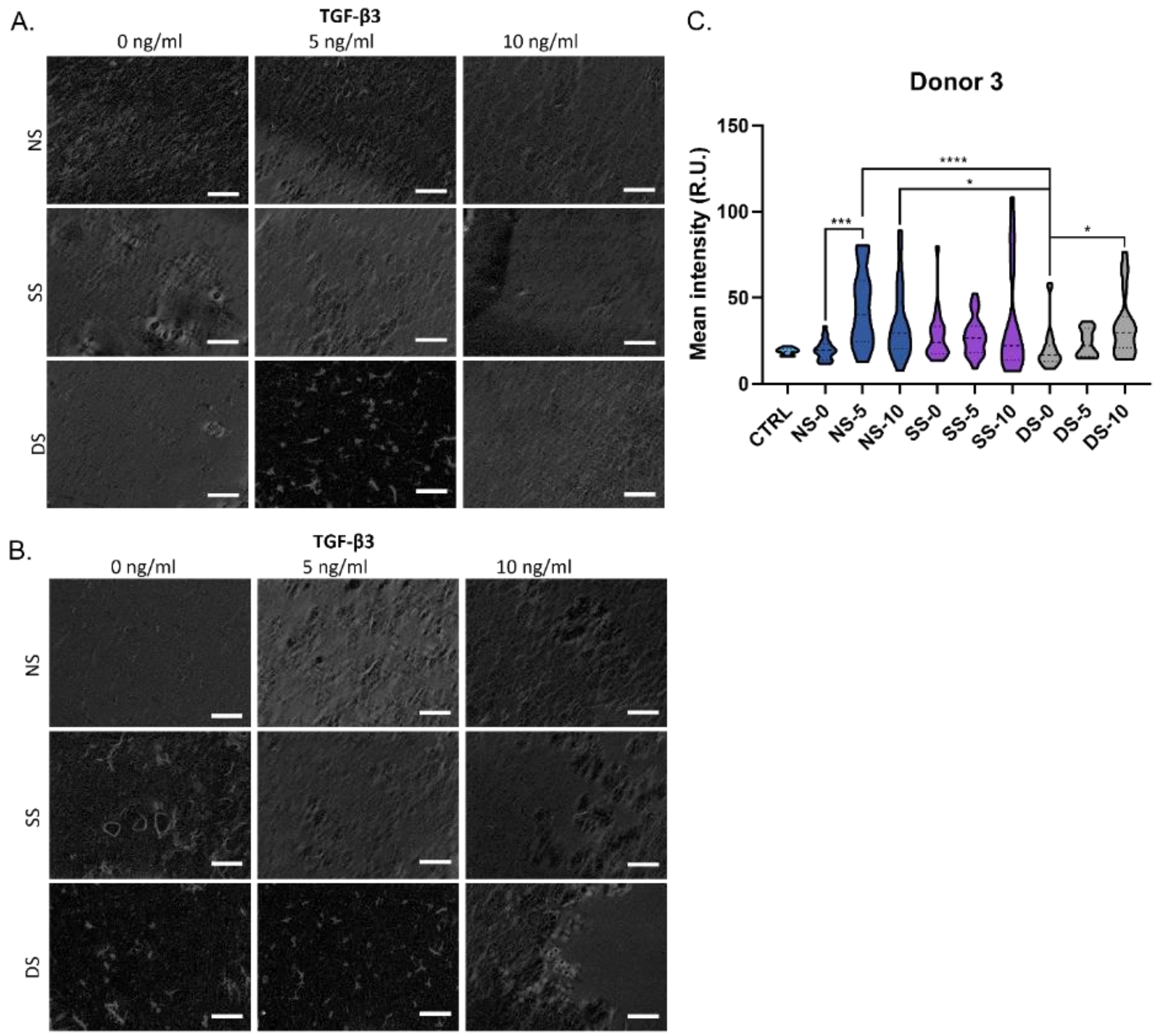

Supplementary Figure 15. Birefringence microscopy images of picrosirius red stained sections near the surface (A) and centre (B) of the hydrogel for Donor 3 (N=3). Collagen fibres appear white against the grey background. Scale bar = 100 µm. (C) Quantitation of birefringence microscopy image intensity of triplicate measures of biological and technical replicates. Graphs are shown truncated violin plots, N = 3; ns, p>0.05; *, p≤0.05; **, p≤0.01; ***, p≤0.001.

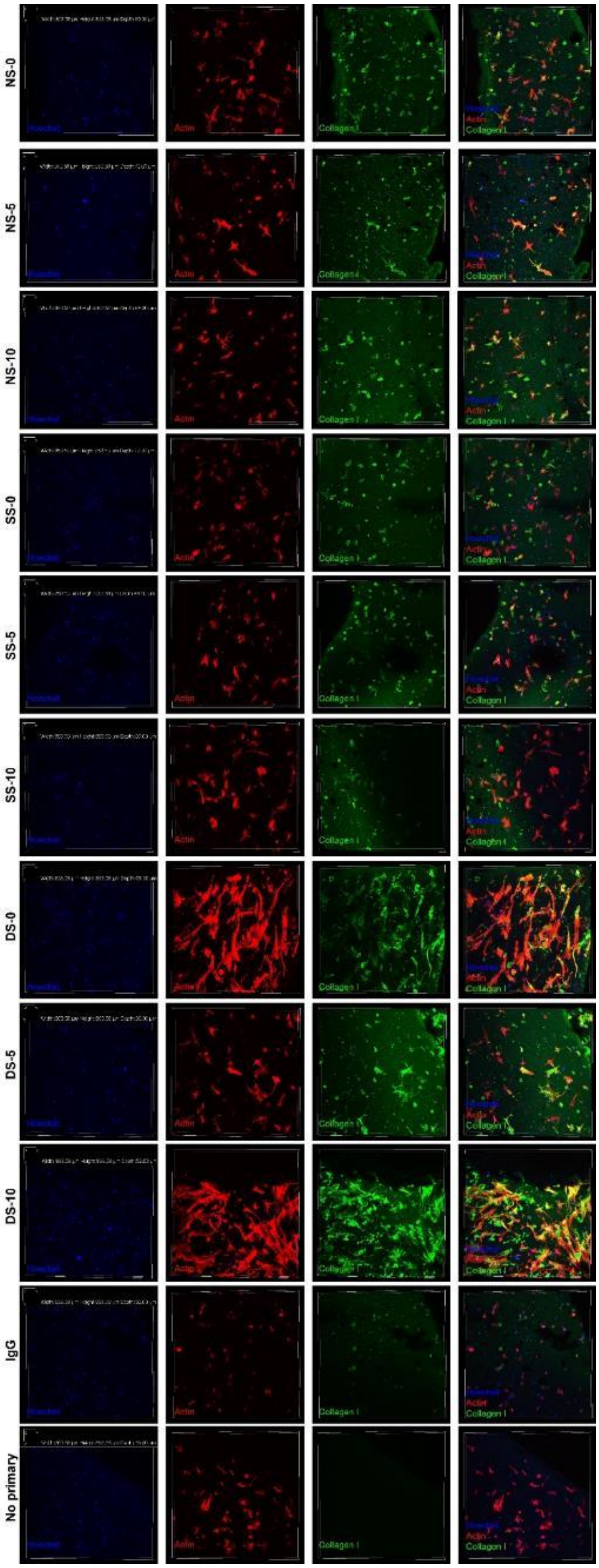

Supplementary Figure 16.

Immunohistochemical staining for collagen type I showing nuclei (blue), actin filaments (red), and collagen type I (green). N=3.
